# Towards the Prediction of Sandwich Composites Durability in Severe Condition of Temperature: A New Numerical Model Describing the Influence of Material Water Content during a Fire Scenario

**DOI:** 10.3390/ma13235420

**Published:** 2020-11-28

**Authors:** Juan Pablo Márquez Costa, Vincent Legrand, Sylvain Fréour, Frédéric Jacquemin

**Affiliations:** Institut de Recherche en Génie Civil et Mécanique (GeM) UMR CNRS 6183, Université de Nantes, Equipe Etat Mécanique et Microstructure des Matériaux (E3M), 58 Rue Michel Ange, BP 420, 44606 Saint-Nazaire CEDEX, France; marquezjpablo@gmail.com (J.P.M.C.); sylvain.freour@univ-nantes.fr (S.F.); frederic.jacquemin@univ-nantes.fr (F.J.)

**Keywords:** sandwich composites, moisture influence, extreme environment, fire resistance, properties prediction, numerical modeling

## Abstract

An advanced fire thermal model was developed to predict the evolution of the temperature and decomposition gradient across a sandwich composite structure when exposed to high temperatures (fire). This model allows the prediction of a large numbers of parameters, such as thermal expansion, gas mass storage, porosity, permeability, density, and internal pressure. The highlight of this model is that we consider, in the sandwich constituents (core and skins), additional parameters, such as changing volume porosities, other coupled constituents (as infused resin in the balsa core), and what make the main originality of the present approach: moisture content (free and bounded water). The time dependence of many parameters, i.e., among others, the combustion advancing front and mechanical properties, can be predicted in a large number of material and fire scenarios. The proposed approach was validated in the case of sandwich panels, with glass/polyester or glass/vinyl ester skins and balsa core, exposed to high temperatures up to 750 °C. The influence of water on the thermal and mechanical responses is also highlighted.

## 1. Introduction

The interest to set a material under extreme conditions is the possibility to analyze it in non-ambient conditions. The material properties could drastically change and new properties could be discovered and studied. This properties variations occur when a material is exposed to a critical environment of high or low temperature [1,2], generally coupled with another stress, such as a light irradiation [3,4], a mechanical stress [5,6], or an intense magnetic or a high-pressure fields [7,8]. Subjected to these extreme environments, the material may undergo drastic chemical and/or physical modifications, inducing phase transitions or metastable states [9,10]. A very interesting goal to study materials behaviors under extreme conditions is to predict and also optimize them under ambient thermodynamic conditions, and use them for new day-to-day applications. Measurements in extreme conditions are both a scientific challenge, to understand the properties of materials, and a technical challenge to study the material in specific and very severe environments. Measurements in extreme conditions present both a technical challenge (analysis in specific severe environments) and also a real scientific challenge to understand the properties and design new materials. Composite materials with an organic matrix analyzed under high temperature conditions are also a very topical subject of study [11,12,13,14,15,16,17,18].

Sandwich composites materials are an extended solution in naval or aircraft structures in order to improve the stiffness-to-weight ratio respected to classical stratified composites and metallic materials [19]. Sandwich plates are typically made of a core covered by thin skins. Composite sandwich consist frequently of a thick lightweight core and thin stiff skins. Main function of the core is to improve the bending, transversal, and compression stiffness, while skins assure the sandwich traction stiffness. This combination makes it possible to manufacture excellent solution concerning lightness, durability, and rigidity. Different solutions can be adapted depending on the application. Carbon fiber reinforced polymer bonded to aramid or aluminum honeycomb cores are usually preferred in the aeronautical and aerospace industries, while glass fiber reinforced polymer combined to light balsa or polymer foam are more frequently used in the marine and naval industry. Nevertheless, in the context of composites subjected to severe environments [11,12,20,21], the understanding of thermal and mechanical response of sandwich materials is necessary for better introducing and optimizing them in structural solutions.

Even if structures made of sandwich composites are recognized to have very good properties, they nevertheless remain sensitive to damage caused by mechanical stress, such as bending, traction, compression, or even an impact. These stresses should cause a drastic drop in their rigidity and play a role in the safety of the structure [22,23]. Several experimental studies make it possible to highlight the modifications of post-event mechanical properties, in particular, the tests of residual compressive strength [24], residual tensile strength [24], 3-point bending, or even impact tests at low speed [24,25,26,27,28]. Coupled with numerical modeling, these analyzes provide information on failure mechanisms and damage modes.

In several temperature or moisture conditions, sandwich can present some disadvantages related to its thermal behavior (such as moderate hear resistance, water absorption, thermal aging [29,30]) and its mechanical behavior (such as cracking and delamination [31]). In addition, the different behavior of skins and core make it difficult to analyze the interaction of physical phenomena across the material thickness. Thermal degradation of sandwich is required to better predicting mechanical performances. Several authors studied the thermal degradation both for laminated and sandwich composites [14,15,20,32,33,34]. However, less works concern the effects of moisture [29,33]. This topic becomes important for sandwich composites, since their constituents exhibit very different and heterogeneous thermal degradation and moisture properties. Particularly, thermal degradation kinetics, moisture expansion coefficients, and maximum moisture absorption capacity are influenced by temperature, especially in the case of porous materials, such as balsa [29,35]. Consequently, both the thermal and mechanical behavior of sandwich composites are strongly dependent on the moisture absorption. It is then mandatory to understand all the physical, chemical, and mechanical phenomena, but also the degradation mechanisms, regarding fire resistance and moisture absorption of sandwich composites.

In this work, we propose an advanced one-dimensional numerical model to predict and analyze the hygro-thermo-mechanical properties of sandwich composite materials (E-glass fibers with vinyl ester or polyester skins surrounding a balsa core) in the case of fire resistance, i.e., in high enough temperature conditions for reaching thermal degradation.

The novelty of our model consists in taking into account the diffusion of water in the sandwich material (both in skins and the combustible core). This moisture particularly affects the durability of the material under ambient conditions. This is even more true in fire resistance condition because of the influence of moisture on the sandwich thermal degradation. We also included a quantity of resin infused in the balsa core to take into account the real manufacturing conditions. These improvements lead to additional difficulties during the programming of the model as a consequence of the high complexity and coupling of the problem. The numerical resolution was implemented in *Mathematica 10* for solving the non-linear system of Partial Differential Equations (PDEs) presented in the next section. The numerical resolution strategy allows us to get interpolation function for each variable to evaluate them as a function of time and though-thickness *x*-coordinate. Thus, unknowns can be evaluated for each constituent of the sandwich composite material, for example, the fact that the water, while evaporating, will cause cavities (variation of porosity), with variations of pressure and temperature, then modifying the thermal response, particularly the material thermal expansion. However, the numerical results are in very good agreement with the experimental results, which makes it possible to really tend towards predictions with a very high rate of reliability.

## 2. Mathematical Formulation

### 2.1. Thermal Model Development

#### 2.1.1. Principle of the Thermal Model

The thermal model of Gibson et al. [15,16] predicts the thermal behaviors of composite materials when subject to high temperatures, describing the most influent thermal processes occurring during a composite degradation when exposed to fire. Based on it, we developed an improved one-dimensional model [21] to simulate the time dependence of the thermal degradation and temperature profiles in the through-thickness direction of composite laminates exposed to constant unidirectional heat flux (which represents a simplification of the fire effects on the material front surface). This last model expresses the heat transfer analyzing phenomena as considered in the literature [14,15,16]. These phenomena are commonly the heat conduction through the material, the thermal conductivity of convective gas transport from the decomposed regions to the hot composite surface, and also the chemical reactions of decomposition that produce or consume heat, depending on the endothermic or exothermic nature of them. This improved model also considers others important phenomena, such as the thermo-chemical deformation in the heat flux direction, internal pressure rise, porosity variation, gas storage, and permeability evolutions. All these later additional phenomena cannot be neglected in order to calculate a more accurate thermal response. However, some assumptions were introduced to simplify this model. They are the following: (i) thermal exchanges between the solid material and decomposition gases occur until thermal equilibrium, (ii) flame modeling during the composite combustion is not taken into account, (iii) initiation and propagation of flame are not considered in the analysis, (iv) decomposition gases behavior is assumed to be ideal, and (v) gas flow is governed by Darcy’s law, and the absence of the decomposition gases reaction is supposed.

Thus, the one-dimensional energy conservation equation we proposed is expressed by Equation (1):

(1) $$\begin{array}{c} \left[m_{s}\left(x,t\right)c_{p}\left(x,t\right)+m_{g}\left(x,t\right)c_{pg}\left(x,t\right)\right]\frac{\partial T\left(x,t\right)}{\partial t}=\frac{\partial }{\partial x}\{[k_{g}\left(x,t\right)\phi \left(x,t\right)+k_{x}\left(x,t\right)(1-\\ \phi \left(x,t\right))]\frac{\partial T\left(x,t\right)}{\partial x}\}\Delta x\left(x,t\right)\Delta A-{\dot{m}}_{g}\left(x,t\right)c_{pg}\left(x,t\right)\frac{\partial T\left(x,t\right)}{\partial x}\Delta x\left(x,t\right)+\\ m_{s}\left(x,t\right)\;A\;{\left[\alpha \left(x,t\right)\right]}^{n}e^{-\frac{E_{a}}{R\;T\left(x,t\right)}}\left[Q_{p}+h_{s}\left(x,t\right)-h_{g}\left(x,t\right)\right]. \end{array}$$

In Equation (1), α(x,t) is virgin material fraction remaining in the solid matrix:

(2) $$\alpha \left(x,t\right)=\frac{m_{m}\left(x,t\right)-m_{mf}}{m_{m0}-m_{mf}},$$

where $m_{m0}$, $m_{m}\left(x,t\right)$, and $m_{mf}$ are, respectively, the initial, instantaneous, and final polymer matrix mass, experimentally determined using the resin decomposition curve obtained by thermo-gravimetric analysis (TGA). α(x,t) is used as a local variable to account for the decomposition process advancement. It makes it possible to distinguish between the instantaneous virgin material fraction during its decomposition. As a consequence, a charred residue (i.e., char) is produced.

In Equation (1), considering a point of interest, *T*(*x*,*t*) is the instantaneous temperature, where *t* is the time, and *x* the through-thickness coordinate, placed through the thickness direction at the distance *x* from the front surface.

The parameters $c_{p}\left(x,t\right)$,$\;c_{pg}\left(x,t\right)$ are specific heat of solid laminate and gases; $m_{s}\left(x,t\right)$, $m_{g}\left(x,t\right)$ are the solid laminate and gases mass; $k_{x}\left(x,t\right)$ and $k_{g}\left(x,t\right)$ are the thermal conductivity of the solid composite and gases, all considered in the direction of thickness. $Q_{p}$ is the polymer matrix endothermic decomposition energy. ${\dot{m}}_{g}\left(x,t\right)$ is the volatile products mass flow. The enthalpies $h_{s}\left(x,t\right)=\int_{T_{\infty }}^{T\left(x,t\right)}c_{p}\left(x,t\right)\;dT$ and $h_{g}\left(x,t\right)=\int_{T_{\infty }}^{T\left(x,t\right)}c_{pg}\left(x,t\right)\;dT$ are, respectively, for the solid laminated composite material and volatiles, with $T_{\infty }$ referring to the ambient temperature.

In Equation (1), one could note the influence of the internal pressure and of the thermal expansion through the porosity ϕ(x,t) and the variable Δx(x,t), respectively.

**Figure 1:**
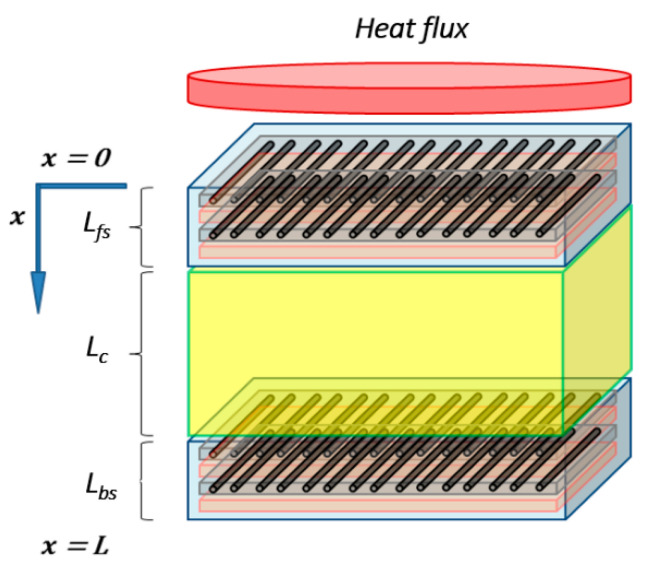
Scheme of thermal model for sandwich composite materials exposed to a constant unidirectional heat flux (balsa core in yellow; polymeric resin in orange; glass fibers in black).

This advanced thermal model, used to analyze laminate composite subject to fire, can be adapted to multi-constituent material. Thus, it was adapted for sandwich materials, made with composite skins (organic matrix and glass fibers) surrounding a combustible core (balsa). This could be done by supposing different material properties in relation to the sandwich constituents and to the position of each constituents inside the whole sandwich structure. The basic energy conservation equation, as proposed by Gibson et al. [15], could so be adapted for an evaluation in the front skin (fs), the core (c), or the back skin (bs), as illustrated in Figure 1. It is noted that interfaces or bonding layers between constituent are not modeled. Its volume degradation is assumed neglected with respect to thermal degradation of main constituents (skins and core) because of high ratios between skins (or core) and interface thickness.

#### 2.1.2. Basics of the Model for Sandwich Composite Materials

A system of energy conservation equations can be expressed as the following Equation (3), using different material properties and kinetic thermal decomposition parameters, depending on the constituent and its local position related to the sandwich material global thickness:

$$\begin{array}{c} \left[m_{s,fs}\left(x,t\right)c_{p,fs}\left(x,t\right)\right]\frac{\partial T\left(x,t\right)}{\partial t}=\frac{\partial }{\partial x}\left\{\left[k_{x,fs}\left(x,t\right)\right]\frac{\partial T\left(x,t\right)}{\partial x}\right\}-{\dot{m}}_{g,fs}\left(x,t\right)c_{pg,fs}\left(x,t\right)\frac{\partial T\left(x,t\right)}{\partial x}+\\ m_{s,fs}\left(x,t\right)\;A_{fs}\;{\left[\alpha_{fs}\left(x,t\right)\right]}^{n_{fs}}e^{-\frac{E_{a,fs}}{R\;T\left(x,t\right)}}\left[Q_{p,fs}+h_{s,fs}\left(x,t\right)-h_{g,fs}\left(x,t\right)\right]\;\mathrm{for}\;0\leq x\leq L_{fs}, \end{array}$$

$$\begin{array}{c} \left[m_{s,c}\left(x,t\right)c_{p,c}\left(x,t\right)\right]\frac{\partial T\left(x,t\right)}{\partial t}=\frac{\partial }{\partial x}\left\{\left[k_{x,c}\left(x,t\right)\right]\frac{\partial T\left(x,t\right)}{\partial x}\right\}-{\dot{m}}_{g,c}\left(x,t\right)c_{pg,c}\left(x,t\right)\frac{\partial T\left(x,t\right)}{\partial x}+\\ m_{s,c}\left(x,t\right)\;A_{c}\;{\left[\alpha_{c}\left(x,t\right)\right]}^{n_{c}}e^{-\frac{E_{a,c}}{R\;T\left(x,t\right)}}\left[Q_{p,c}+h_{s,c}\left(x,t\right)-h_{g,c}\left(x,t\right)\right]\;\mathrm{for}\;L_{fs}\leq x\leq L_{fs}+L_{c}, \end{array}$$

(3) $$\begin{array}{c} \left[m_{s,bs}\left(x,t\right)c_{p,bs}\left(x,t\right)\right]\frac{\partial T\left(x,t\right)}{\partial t}=\frac{\partial }{\partial x}\left\{\left[k_{x,bs}\left(x,t\right)\right]\frac{\partial T\left(x,t\right)}{\partial x}\right\}-{\dot{m}}_{g,bs}\left(x,t\right)c_{pg,bs}\left(x,t\right)\frac{\partial T\left(x,t\right)}{\partial x}+\\ m_{s,bs}\left(x,t\right)\;A_{bs}\;{\left[\alpha_{bs}\left(x,t\right)\right]}^{n_{bs}}e^{-\frac{E_{a},bs}{R\;T\left(x,t\right)}}\left[Q_{p,bs}+h_{s,bs}\left(x,t\right)-h_{g,bs}\left(x,t\right)\right]\;\mathrm{for}\;L_{fs}+L_{c}\leq x\leq L_{fs}+L_{c}+L_{bs}. \end{array}$$

Following this approach, one could obtain the following systems of equations of heat transfer for sandwich composites, considering the improved energy conservation equation for each constituent introduced in Equation (1). Thus, the final system is expressed as follows:

$$\begin{array}{c} \left[m_{s,fs}\left(x,t\right)c_{p,fs}\left(x,t\right)+m_{g,fs}\left(x,t\right)c_{pg,fs}\left(x,t\right)\right]\frac{\partial T\left(x,t\right)}{\partial t}=\frac{\partial }{\partial x}\{[k_{g,fs}\left(x,t\right)\phi_{fs}\left(x,t\right)+\\ k_{x,fs}\left(x,t\right)\left(1-\phi_{fs}\left(x,t\right)\right)]\frac{\partial T\left(x,t\right)}{\partial x}\}\Delta x_{fs}\left(x,t\right)\Delta A_{fs}-\\ {\dot{m}}_{g,fs}\left(x,t\right)c_{pg,fs}\left(x,t\right)\frac{\partial T\left(x,t\right)}{\partial x}\Delta x_{fs}\left(x,t\right)+m_{s,fs}\left(x,t\right)\;A_{fs}\;{\left[\alpha_{fs}\left(x,t\right)\right]}^{n_{fs}}e^{-\frac{E_{a,fs}}{R\;T\left(x,t\right)}}[Q_{p,fs}+\\ h_{s,fs}\left(x,t\right)-h_{g,fs}\left(x,t\right)]\;\mathrm{for}\;0\leq x\leq L_{fs}, \end{array}$$

$$\begin{array}{c} {}[m_{s,c}\left(x,t\right)c_{p,c}\left(x,t\right)+m_{g,c}\left(x,t\right)c_{pg,c}\left(x,t\right)]\frac{\partial T\left(x,t\right)}{\partial t}=\frac{\partial }{\partial x}\{[k_{g,c}\left(x,t\right)\phi_{c}\left(x,t\right)+\\ k_{x,c}\left(x,t\right)\left(1-\phi_{c}\left(x,t\right)\right)]\frac{\partial T\left(x,t\right)}{\partial x}\}\Delta x_{c}\left(x,t\right)\Delta A_{c}-{\dot{m}}_{g,c}\left(x,t\right)c_{pg,c}\left(x,t\right)\frac{\partial T\left(x,t\right)}{\partial x}\Delta x_{c}\left(x,t\right)+\\ m_{s,c}\left(x,t\right)\;A_{c}\;{\left[\alpha_{c}\left(x,t\right)\right]}^{n_{c}}e^{-\frac{E_{a,c}}{R\;T\left(x,t\right)}}\left[Q_{p,c}+h_{s,c}\left(x,t\right)-h_{g,c}\left(x,t\right)\right]\;\mathrm{for}\;L_{fs}\leq x\leq L_{fs}+L_{c}, \end{array}$$

(4) $$\begin{array}{c} \left[m_{s,bs}\left(x,t\right)c_{p,bs}\left(x,t\right)+m_{g,bs}\left(x,t\right)c_{pg,bs}\left(x,t\right)\right]\frac{\partial T\left(x,t\right)}{\partial t}=\frac{\partial }{\partial x}\{[k_{g,bs}\left(x,t\right)\phi_{bs}\left(x,t\right)+\\ k_{x,bs}\left(x,t\right)\left(1-\phi_{bs}\left(x,t\right)\right)]\frac{\partial T\left(x,t\right)}{\partial x}\}\Delta x_{bs}\left(x,t\right)\Delta A_{bs}-{\dot{m}}_{g,bs}\left(x,t\right)c_{pg,bs}\left(x,t\right)\frac{\partial T\left(x,t\right)}{\partial x}\Delta x_{bs}\left(x,t\right)+\\ m_{s,bs}\left(x,t\right)\;A_{bs}\;{\left[\alpha_{bs}\left(x,t\right)\right]}^{n_{bs}}e^{-\frac{E_{a,bs}}{R\;T\left(x,t\right)}}\left[Q_{p,bs}+h_{s,bs}\left(x,t\right)-h_{g,bs}\left(x,t\right)\right]\;\mathrm{for}\;L_{fs}+L_{c}\leq x\leq L_{fs}+\\ L_{c}+L_{bs}. \end{array}$$

As it is mentioned above, a crucial point in the writing of the thermal model for a sandwich composite material is to consider the position of each constituent in the global sandwich structure to appropriately evaluate the thermal behavior at each coordinate of the thickness. As it seems evident, mass degradation will start at front skin and progresses as a function of time and of the through-thickness direction, being careful that the heat flux could also be sufficiently high to simultaneously activate pertinent degradation reactions of the front skin, the core, and/or the back skin.

The present results suppose simple cases of decomposition kinetics, expressed by single step reactions for each constituent individually, but easily adapted to simulate different steps of decomposition for each constituent depending on the temperature conditions. For example, fibers degradation could be envisaged for composite laminates submitted to very high flux, as described elsewhere [17,18]. Resin pyrolysis reactions of the front and back skins, as the balsa core pyrolysis reaction, are modeled in term of mass following typical Arrhenius laws:

$$\frac{1}{m_{m0,fs}-m_{mf,fs}}\frac{\partial m_{m,fs}\left(x,t\right)}{\partial t}=-A_{fs}\;{\left[\alpha_{fs}\left(x,t\right)\right]}^{n_{fs}}e^{-\frac{E_{a},fs}{R\;T\left(x,t\right)}}\;\mathrm{for}\;0\leq x\leq L_{fs},$$

$$\frac{1}{m_{m0,c}-m_{mf,c}}\frac{\partial m_{m,c}\left(x,t\right)}{\partial t}=-A_{c}\;{\left[\alpha_{c}\left(x,t\right)\right]}^{n_{c}}e^{-\frac{E_{a},c}{R\;T\left(x,t\right)}}\;\mathrm{for}\;L_{fs}\leq x\leq L_{fs}+L_{c},$$

(5) $$\frac{1}{m_{m0,bs}-m_{mf,bs}}\frac{\partial m_{m,bs}\left(x,t\right)}{\partial t}=-A_{fs}\;{\left[\alpha_{bs}\left(x,t\right)\right]}^{n_{bs}}e^{-\frac{E_{a},\;bs}{R\;T\left(x,t\right)}}\;\mathrm{for}\;L_{fs}+L_{c}\leq x\leq L_{fs}+L_{c}+L_{bs}.$$

A is the reaction rate constant, $E_{a}$ is the activation energy, and n is the reaction order. *A*, *E_a_*, and *n* are obtained by TGA during the decomposition reaction of the skins and core materials. This formulation expressed in Equation (5) implies that the temperature evolutions and mass loss profiles are different as a function of time for the front and back skins. This could be easily understood as the front skin is directly subject to the heat flux, whereas the back skin is protected by the above layers (front skin and core).

#### 2.1.3. Thermal Expansion

In addition, PDEs are formulated for the through-thickness thermal expansion (Equation (6)) and the material permeability variation (Equation (7)), following a typical approach based on rules of mixture in order to take into account for virgin material and char contributions [17,32,36]. Equations (6) and (7) are expanded for each constituent (skins, core) of the sandwich composite material, depending on the though-thickness *x*-coordinate:

(6) $$\frac{1}{L_{0}}\frac{\partial L\left(x,t\right)}{\partial t}=\alpha_{v}\;\alpha \left(x,t\right)\frac{\partial T\left(x,t\right)}{\partial t}+\alpha_{c}\left[1-\alpha \left(x,t\right)\right]\frac{\partial T\left(x,t\right)}{\partial t}+\frac{\eta }{m_{m0}}\frac{\partial m_{m}\left(x,t\right)}{\partial t},$$

(7) $$\frac{1}{\gamma_{0}}\frac{\partial \gamma \left(x,t\right)}{\partial t}=\psi_{v}\;\alpha \left(x,t\right)\frac{\partial T\left(x,t\right)}{\partial t}+\psi_{c}\left[1-\alpha \left(x,t\right)\right]\frac{\partial T\left(x,t\right)}{\partial t}+\frac{\zeta }{m_{m0}}\frac{\partial m_{m}\left(x,t\right)}{\partial t},$$

with $\alpha_{v}$ and $\alpha_{c}$ the linear expansion coefficient, and $\psi_{v}$ and $\psi_{c}$ the permeability coefficient, of virgin (*v*) and char (*c*) materials, respectively. Equations (6) and (7) contain non-dimensional factors: the expansion term η and the permeability term ζ as a consequence of the solid matrix pyrolytic decomposition.$\;\gamma_{0}$ is the initial permeability. All the parameters are different for each constituent of the sandwich composite, allowing to evaluate the instantaneous thickness L(x,t) and the instantaneous permeability γ(x,t) through the thickness *x*-coordinate for each constituent.

#### 2.1.4. Gases Flow and Pressure

The gas storage in each skin solid matrix and in the core, as well as the gas mass flux of each constituent of the sandwich material, are calculated according to the mass conservation equation, which is simplified as follows [37]:

(8) $$-\frac{\partial m_{s}\left(x,t\right)}{\partial t}=\frac{\partial m_{g}\left(x,t\right)}{\partial t}+\frac{\partial {\dot{m}}_{g}\left(x,t\right)}{\partial t}\Delta x\left(x,t\right).$$

Equation (8) is evaluated for each constituent of the sandwich structure depending on the *x*-coordinate. It models the transport phenomena of decomposition gas through the material thickness, related to the decomposition rate modeled as a typical Arrhenius expression (Equation (5)) for each constituent of the sandwich composite.

The transport speed of the mass flow of gases ${\dot{m}}_{g}\left(x,t\right)$ can be expressed using the Darcy’s expression given by Equation (9), whereas the pressure P(x,t) can be expressed using the ideal gas state given by Equation (10) [32]:

(9) $${\dot{m}}_{g}\left(x,t\right)=-\frac{\gamma \left(x,t\right)\;m_{g}\left(x,t\right)}{\mu \;\phi \left(x,t\right)\;\Delta x\left(x,t\right)}\frac{\partial P\left(x,t\right)}{\partial x},$$

(10) $$P\left(x,t\right)=\frac{m_{g}\left(x,t\right)\;R\;T\left(x,t\right)}{M\;\phi \left(x,t\right)\;\Delta x\left(x,t\right)},$$

with μ the viscosity of the decomposition gases; *R* is the ideal gases constant; *M* is the decomposition gases average molecular weight, which might be different for each constituent but assumed equal to simplify the problem in the case of similar volatile products for each constituent of the sandwich material.

As for laminated composite materials, the pressure influence due to the gas storage in each constituent is indirectly considered implicitly in the heat equation for each constituent of the sandwich expressed by Equation (4), of which terms of conductivity heat transfer are modified by the porosity depending on the through-thickness coordinate of each constituent.

#### 2.1.5. Thermal Parameters

Thermal properties are estimated for each sandwich composite constituent, and we chose a modeling based on homogenization laws, to consider the spatial and time advancement of the pyrolysis reaction through the parameter α(x,t) [12,21]. Then, the composite material conductivity, Equation (11), and the specific heat, Equation (12), can be modeled during fire decomposition using the different states (virgin or char) of the different sandwich constituents (front skin, core, and back skin):

(11) $$k_{x}\left(x,t\right)=\alpha \left(x,t\right)\;k_{v}\left(T\left(x,t\right)\right)+\left[1-\alpha \left(x,t\right)\right]\;k_{c}\left(T\left(x,t\right)\right),$$

(12) $$c_{p}\left(x,t\right)=\alpha \left(x,t\right)\;c_{pv}\left(T\left(x,t\right)\right)+\left[1-\alpha \left(x,t\right)\right]\;c_{pc}\left(T\left(x,t\right)\right),$$

where $k_{v}\left(T\right)$ and $k_{c}\left(T\right)$ are the thermal conductivities, and $\;c_{pv}\left(T\right)$ and $\;c_{pc}\left(T\right)$ are the specific heats, of the virgin and char materials, respectively, which take different expression depending on the considered constituent.

**Figure 2:**
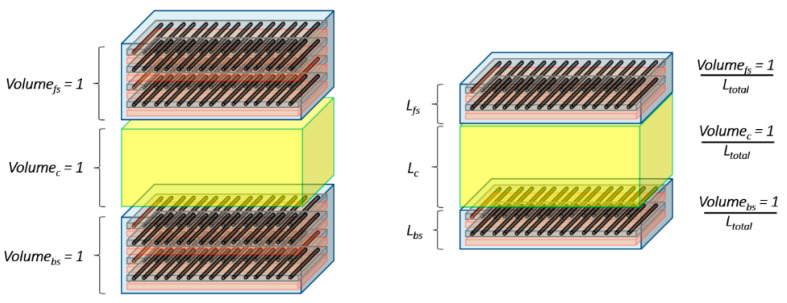
Scheme of the conversion of unit control volumes to real volumes in order to calculate the global mass of a composite sandwich material.

In this model, it is considered unit control volumes for each constituent of the sandwich material (front skin, core, and back skin). Therefore, both the evolution of the temperature T(x,t) and the instantaneous mass of each constituent are calculated separately. The overall instantaneous mass loss and the remaining mass of the sandwich structure during fire exposure can thus be estimated. As shown in Figure 2, unit volumes should be converted to real volumes, relatively to the thickness of each material, in order to calculate the mass contribution of each component to the overall mass of the sandwich having a total thickness $L_{total}$.

The instantaneous global mass of the sandwich can be normalized to the initial mass of the sandwich $m_{sandwich0},$ in order to formulate the overall weight loss of the sandwich. The expression of the global remaining mass fraction of the sandwich material $RMF_{sandwich}\left(x,t\right)$ over time and as a function of *x*-coordinate is given by Equation (13):

(13) $$RMF_{sandwich}\left(x,t\right)=\frac{m_{fs}\left(x,t\right)\;L_{fs}+m_{c}\left(x,t\right)\;L_{c}+m_{bs}\left(x,t\right)\;L_{bs}}{m_{sandwich0}\;L_{total}}.$$

#### 2.1.6. Initial and Boundary Conditions

Initial and boundary conditions are established to make the thermal problem numerically solvable, and it is important to note that boundary conditions are established for the whole sandwich, which means for the upper surface of the front skin and for the lower surface of the back skin. The initial conditions are:

$$\begin{array}{c} T\left(x,0\right)=T_{\infty },\;m_{m\left(fs,c,bs\right)}\left(x,0\right)=m_{m0\left(fs,c,bs\right)},\;{\dot{m}}_{g\left(fs,c,bs\right)}\left(x,0\right)=0\\ \mathrm{for}\;0\leq x\leq L_{total}, \end{array}$$

(14) $$\begin{array}{c} m_{g\left(fs,c,bs\right)}\left(x,0\right)=m_{g0\left(fs,c,bs\right)},\;P\left(x,0\right)=P_{0},\;L_{\left(fs,c,bs\right)}\left(x,0\right)=L_{0\left(fs,c,bs\right)}\\ \mathrm{for}\;0\leq x\leq L_{total}. \end{array}$$

The heat transfer are principally due to radiative mechanisms [17,32,37], but it must also consider the thermal convection of the heating source. The Stefan-Boltzmann law is then written as follows:

(15) $$\begin{array}{c} -\left[k_{g,fs}\left(x,t\right)\phi_{fs}\left(x,t\right)+k_{x,fs}\left(x,t\right)\left(1-\phi_{fs}\left(x,t\right)\right)\right]{\frac{\partial T\left(x,t\right)}{\partial x}|}_{x=0}=[\varepsilon_{s}\;q_{radiation}^{\prime \prime }-\\ \sigma \;\varepsilon_{msup,fs}T^{4}\left(x,t\right)]+h_{convsup,fs}\left[T_{\infty }-T\left(x,t\right)\right]\;\mathrm{for}\;x=0\;\mathrm{and}\;\forall t>0. \end{array}$$

In Equation (15), right-hand side represents the net surface heat flux on the composite [20]. It is the net heat flux of the source that is received by the frontal surface of the composite which is the combination of the radiative and convective contributions. $\varepsilon_{s}$ is the emissivity of the source. $\varepsilon_{msup,fs}$ and $h_{convsup,fs}$ are the emissivity and the convection coefficient of the upper surface of the front skin of the sandwich composite material, respectively. σ is Stefan-Boltzmann’s constant.

Boundary condition on the lower surface of the back skin of the sandwich composite $\left(x=L_{total}\right)$, surface not exposed to the heat source, is also written as the sum of radiative and convective contributions:

(16) $$\begin{array}{c} -\left[k_{g,bs}\left(x,t\right)\phi_{bs}\left(x,t\right)+k_{x,bs}\left(x,t\right)\left(1-\phi_{bs}\left(x,t\right)\right)\right]{\frac{\partial T\left(x,t\right)}{\partial x}|}_{x=L_{total}}=\\ \sigma \;\varepsilon_{minf,bs}\left[T^{4}\left(x,t\right)-T_{\infty }^{4}\right]+h_{convinf,bs}\left[T\left(x,t\right)-T_{\infty }\right]\;\mathrm{for}\;x=\;L_{total}\;\mathrm{and}\;\forall t>0, \end{array}$$

where $\varepsilon_{minf,bs}$ is the emissivity, and $h_{convinf,bs}$ is the heat convection coefficients at the back surface of the lower skin of the sandwich composite material. Both coefficients are different for the cold and hot surfaces, even if the composite material is the same for both skins. These parameters are functions of the temperature T(x,t) and of the decomposition degree α(x,t) of each constituent. However, these variations are quite small and often considered as constant values for the two surfaces of each skin.

Others boundary conditions at $\left(x=L_{total}\right)$ (i.e., back surface) are necessary to close the thermal problem:

(17) $${\dot{m}}_{g,bs}\left(L_{total},t\right)=0,\;P\left(L_{total},t\right)=P_{0}\;\mathrm{for}\;x=L_{total}\;\mathrm{and}\;\forall t>0,$$

with ${\dot{m}}_{g}\left(x,t\right)$ the mass flow of gases, and P(x,t) the internal pressure.

#### 2.1.7. Implementation of the Thermal Model

The thermo-chemical degradation of sandwich composite materials with organic matrix and combustible core is well defined by the set of Equations (4)–(17). All variables and parameters involved in these equations are instantaneous local magnitudes during exposure time to fire and function of the *x*-coordinate in the through-thickness direction. So, they are all coupled with each other and depend on the different properties of each constituent of the sandwich composite material. In order to avoid a large number of equations to solve, a numerical strategy was proposed consisting in defining the different material properties for each constituent as a function of the through-thickness coordinate. It means that a considered property for a given constituent only depends on the *x*-coordinate. Thus, the sandwich thermal problem has the same number of equations as the problem already solved for a laminate composite [21]. The mathematical resolution is then similar and described by a non-linear system of Partial Differential Equations (PDEs) [38]. More details about the numerical method implemented in *Mathematica 10* can be found in J.P. Márquez Costa et al. [21].

The numerical model presented in Section 2.1 is formulated for the whole sandwich taking into account for different properties of constituents through the x-coordinate. Global resolution of the system considering boundary conditions applied on the whole sandwich as presented in Section 2.1.6 allows to get a continuous temperature and global mass loss, even at the constituent transitions. Thus, the numerical resolution of the model assumes ideal no thickness interface between constituents, where evaluated variables are continuous.

### 2.2. Post-Combustion Mechanical Response

The estimation of the post-combustion mechanical properties (i.e., when the material returns to room temperature again) is crucial to appreciate the residual mechanical integrity of a structure made by the studied material. Three point bending flexural test is an accurate experimental method employed to investigate that response [39,40]. The flexural modulus variation as a function of the degradation time is determined. From a numerical point of view, residual mechanical properties are time and temperature dependent, and depend also of the material mass loss through the term α(x,t) implemented in the thermal model. However, they can be calculated in a first approximation decoupled from the thermal model equations, through the results obtained from the evaluation of the thermal model, assuming that degradation of mechanical properties does not significantly change the thermal response.

**Figure 3:**
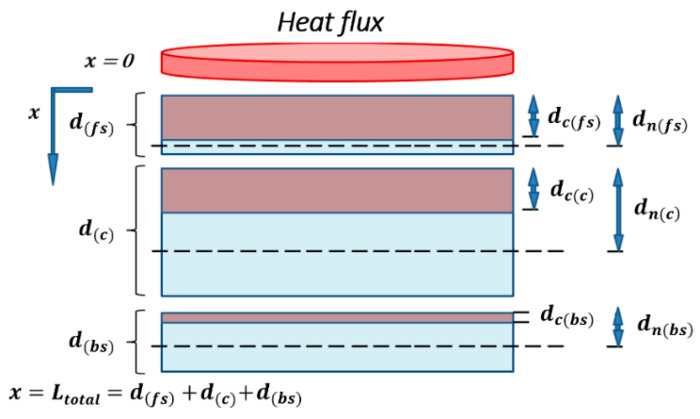
Scheme of the idealized two-layer approach for each constituent in order to study the post-combustion mechanical properties of a sandwich composite material. The decomposed constituent material (char material, in brown) and virgin material (in blue) evolve as a function of time and temperature through the specimen thickness direction *x* (combustion advancing front).

For numerical computation of the normalized flexural modulus for sandwich composite, we assume that each constituent (front skin, core, and back skin) of the sandwich material thermally aged can be represented by a two-layer approach, as shown in Figure 3. The thermo-mechanical model based on a two-layer model for composite laminates [39,40] was used in order to predict the residual mechanical properties evolution. The first layer is composed of char (in brown in Figure 3) due to the material decomposition, in which mechanical properties are weak. The following layer is formed by the virgin material (in blue in Figure 3), in which mechanical properties are the originally one. Between the two layers, char and virgin material, a boundary moves as a function of the combustion time. That boundary represents the combustion advancing front of each constituent, which are directly related to their degree of degradation through the parameter α(x,t), which must be evaluated for the different constituents depending on the through-thickness *x*-coordinate of interest. Thus, the combustion advancing fronts of each constituent are related to each other.

In Figure 3, $d_{\left(fs,c,bs\right)}$ represents the thickness of each constituent (front skin, core, and back skin, respectively) of the sandwich sample. $d_{c\left(fs,c,bs\right)}$ is the carbonized layer thickness of each constituent. $d_{n\left(fs,c,bs\right)}$ is the thickness from the frontal surface to the neutral axis of each constituent material, related to the thicknesses $d_{\left(fs,c,bs\right)}$ and $d_{c\left(fs,c,bs\right)}$. The key parameter to predict the post-combustion properties of each constituent and of the sandwich material is the thickness $d_{c\left(fs,c,bs\right)}$ evolving as a function of each constituent degradation through the term α(x,t), also depending on the fire exposure time and on the others constituent material degradations.

The post-combustion mechanical model allow to evaluate the loss of rigidity of the whole sandwich, through the parameter $\alpha_{\left(fs,c,bs\right)}\left(x,t\right)$ evaluating the level of degradation for each constituent (front skin, core, and back skin). Expression of the flexural modulus 〈EI〉 of composite laminates [39,40] can be extended to the case of each constituent of sandwich composite materials:

$$\begin{array}{c} {\langle EI\rangle }_{fs}=\{\frac{4{\left[d_{\left(fs\right)}-d_{n\left(fs\right)}\left(x,t\right)\right]}^{3}+4{\left[d_{n\left(fs\right)}\left(x,t\right)-d_{c\left(fs\right)}\left(x,t\right)\right]}^{3}}{d_{\left(fs\right)}^{3}}+\\ 4\frac{E_{c\left(fs\right)}}{E_{0\left(fs\right)}}\frac{{\left[d_{n\left(fs\right)}\left(x,t\right)\right]}^{3}-{\left[d_{n\left(fs\right)}\left(x,t\right)-d_{c\left(fs\right)}\left(x,t\right)\right]}^{3}}{d_{\left(fs\right)}^{3}}\}{\langle EI\rangle }_{0\left(fs\right)}, \end{array}$$

$$\begin{array}{c} {\langle EI\rangle }_{c}=\{\frac{4{\left[d_{\left(c\right)}-d_{n\left(c\right)}\left(x,t\right)\right]}^{3}+4{\left[d_{n\left(c\right)}\left(x,t\right)-d_{c\left(c\right)}\left(x,t\right)\right]}^{3}}{d_{\left(c\right)}^{3}}+\\ 4\frac{E_{c\left(\;c\right)}}{E_{0\left(\;c\right)}}\frac{{\left[d_{n\left(\;c\right)}\left(x,t\right)\right]}^{3}-{\left[d_{n\left(\;c\right)}\left(x,t\right)-d_{c\left(c\right)}\left(x,t\right)\right]}^{3}}{d_{\left(c\right)}^{3}}\}{\langle EI\rangle }_{0\left(c\right)}, \end{array}$$

(18) $$\begin{array}{c} {\langle EI\rangle }_{bs}=\{\frac{4{\left[d_{\left(bs\right)}-d_{n\left(bs\right)}\left(x,t\right)\right]}^{3}+4{\left[d_{n\left(bs\right)}\left(x,t\right)-d_{c\left(bs\right)}\left(x,t\right)\right]}^{3}}{d_{\left(bs\right)}^{3}}+\\ 4\frac{E_{c\left(bs\right)}}{E_{0\left(bs\right)}}\frac{{\left[d_{n\left(bs\right)}\left(x,t\right)\right]}^{3}-{\left[d_{n\left(bs\right)}\left(x,t\right)-d_{c\left(bs\right)}\left(x,t\right)\right]}^{3}}{d_{\left(bs\right)}^{3}}\}{\langle EI\rangle }_{0\left(bs\right)}, \end{array}$$

$$d_{n\left(fs\right)}\left(x,t\right)=\frac{d_{\left(fs\right)\;}^{2}E_{0\left(fs\right)}-d_{c\left(fs\right)}^{2}\left(x,t\right)\left[E_{0\left(fs\right)}-E_{c\left(fs\right)}\right]}{2d_{\left(fs\right)}E_{0\left(fs\right)}+2d_{c\left(fs\right)}\left(x,t\right)E_{c\left(fs\right)}-2d_{c\left(fs\right)}\left(x,t\right)E_{0\left(fs\right)}},$$

$$d_{n\left(c\right)}\left(x,t\right)=\frac{d_{\left(c\right)\;}^{2}E_{0\left(fs\right)}-d_{c\left(c\right)}^{2}\left(x,t\right)\left[E_{0\left(c\right)}-E_{c\left(c\right)}\right]}{2d_{\left(c\right)}E_{0\left(c\right)}+2d_{c\left(c\right)}\left(x,t\right)E_{c\left(c\right)}-2d_{c\left(c\right)}\left(x,t\right)E_{0\left(c\right)}},$$

(19) $$d_{n\left(bs\right)}\left(x,t\right)=\frac{d_{\left(bs\right)\;}^{2}E_{0\left(bs\right)}-d_{c\left(bs\right)}^{2}\left(x,t\right)\left[E_{0\left(bs\right)}-E_{c\left(bs\right)}\right]}{2d_{\left(bs\right)}E_{0\left(bs\right)}+2d_{c\left(bs\right)}\left(x,t\right)E_{c\left(bs\right)}-2d_{c\left(bs\right)}\left(x,t\right)E_{0\left(bs\right)}},$$

with $E_{0}$ and $E_{c}$ the initial and final Young’s moduli of the virgin (*_0_*) and char (*c*) materials, respectively, which must be measured for each constituent of the sandwich composite (front skin, core, and back skin). I is the section quadratic moment of the analyzed constituent of the sandwich sample, and ${\langle EI\rangle }_{0}$ the initial flexural modulus of each one. The previous expressions can be simplified assuming $E_{0\left(fs,c,bs\right)}\gg E_{c\left(fs,c,bs\right)}$ (or even $E_{c\left(fs,c,bs\right)}=0$) allowing to obtain elementary expressions as:

(20) $${\langle EI\rangle }_{\left(fs,c,bs\right)}=4\left\{\frac{{\left[d_{\left(fs,c,bs\right)}-d_{n\left(fs,c,bs\right)}\left(x,t\right)\right]}^{3}+{\left[d_{n\left(fs,c,bs\right)}\left(x,t\right)-d_{c\left(fs,c,bs\right)}\left(x,t\right)\right]}^{3}}{d_{\left(fs,c,bs\right)}^{3}}\right\}{\langle EI\rangle }_{0\left(fs,c,bs\right)},$$

(21) $$d_{n\left(fs,c,bs\right)}\left(x,t\right)=\frac{d_{\left(fs,c,bs\right)}^{2}-d_{c\left(fs,c,bs\right)}^{2}\left(x,t\right)}{2\left[d_{\left(fs,c,bs\right)}-2d_{c\left(fs,c,bs\right)}\left(x,t\right)\right]}.$$

Equation (20) allows to immediately calculate the dependence of the flexural modulus as a function of the time and though-thickness coordinate, this for each constituent of the sandwich sample (front skin, core, and back skin) taking into account the induced thermal degradation through the advancing front of each one, and knowing the values of their quadratic moments. Flexural moduli calculation of the whole sandwich sample must be done related to neutral section of the global sample, which implies the application of Steiner’s theorem with respect to the neutral axis of the sandwich material, which coincides with the gravitational mass central section of the sandwich (i.e., central section of core in the case of symmetric sandwich composites). An equivalent flexural modulus in the case of symmetric sandwich composites at ambient temperature can be calculated [41] as the addition of the flexural modulus of every constituent (skins and core):

(22) $${\langle EI\rangle }_{eq}=E_{core}I_{core}+2\left(E_{skin}I_{skin}^{*}\right).$$

Equation (22) can be expanded in order to take into account the contribution of inertial terms of the skins applying the Steiner’s theorem to calculate the equivalent flexural modulus of symmetric sandwich composites:

(23) $${\langle EI\rangle }_{eq}=E_{core}I_{core}+2E_{skin}\left(I_{skin}+S_{skin}d_{m}^{2}\right),$$

with $S_{skin}$ referring to the skin section area, and $d_{m}$ representing the thickness between the middle of a skin and the middle of the core. In the case of sandwich composites exposed to fire, the thermal degradation of each constituent (skins and core) is established independently, and can be thus coupled successively or simultaneously between each other. That induces a different value of $E_{skin}$ for the front and back skins (index *fs* and *bs*, respectively, in Equation (24)). Consequently, Equation (23) is modified as follows for a symmetric sandwich composite material exposed to fire:

(24) $$\begin{array}{c} {\langle EI\rangle }_{eq}\left(x,t\right)=E_{core}\left(x,t\right)I_{core}+\lfloor E_{skin}\left(x,t\right){\left[I_{skin}+S_{skin}d_{m}^{2}]\right\rfloor }_{fs}+\lfloor E_{skin}\left(x,t\right)[I_{skin}+\\ {S_{skin}d_{m}^{2}]\rfloor }_{bs}. \end{array}$$

## 3. Results

### 3.1. Thermal Degradation Prediction

The thermal model exposed in the previous section was used to simulate the thermal degradation of two different sandwich composite materials with organic matrix and combustible balsa core. In the following, numerical results are confronted with experimental data to probe and check the validity of our advanced thermal model. The goal is to use it to predict the thermal behavior of any sandwich composites principally in terms of temperature profiles and mass loss evolution.

#### 3.1.1. Glass/Polyester/Balsa Sandwich Composite

**Figure 4:**
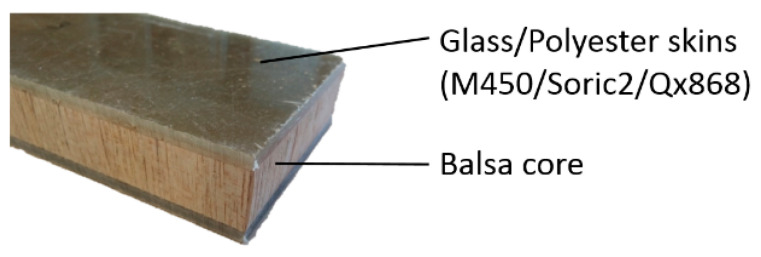
The E-glass/polyester/balsa sandwich composite specimen.

The chosen material is a balsa wood core surrounded by glass/polyester skins (Figure 4). These latter are constituted by M450 (fiberglass chopped strand mat), Soric2 (flow media for resin infusion), QX868 (quadriaxial (0°/−45°/90°/+45°) E-glass draped layer) superposed layers and embedded in polyester resin. Total volume content of fibers is 21%. The thicknesses are 2.5 mm for the front skin, 2.0 mm for the back skin, and14 mm balsa core. Material properties for skins and core are summarized in Table 1.

**Figure 5:**
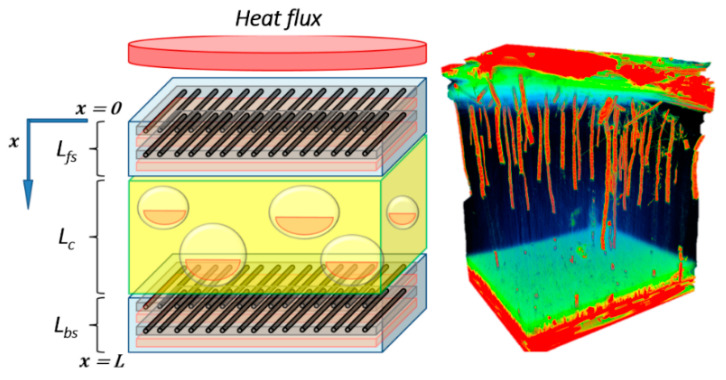
Scheme of thermal model for sandwich composites exposed to a constant unidirectional heat flux considering resin diffusion inside the balsa: each sandwich core volume element is constituted by balsa material + porosity (air) + resin (skin polymeric matrix). μ-tomographic 3D-image reconstruction obtained on a for E-glass/polyester/balsa sandwich composite sample (XRADIA instrument, 15 s exposure time, pixel size 12.6 μm, 721 images).

With the set of Equations (4)–(10) and the initial and boundary conditions, Equations (14)–(17), it is possible to solve the thermal degradation problem of sandwich composite materials exposed to a constant unidirectional heat flux. However, due to the porous balsa core and to the manufacturing process, we took into account in the numerical resolution a certain amount of infused resin inside the balsa core. This is an important highlight inputted in our advanced thermal model, which has, to our knowledge, never been considered before in thermal models relating to composite sandwich materials. Its modeling is based on considering this amount of resin as a new core constituent, modeling it following its associated degradation kinetics. In this way, a coupling between infused resin and balsa core degradation is modeled. Otherwise, when any infused resin is considered, the balsa core degradation would be advanced and the global mass loss evolution would be lightly overestimated, particularly affecting the estimation of the downstream thermal response. Thus, taking into account this infused resin makes it possible to improve the thermal predictive response of the materials, in particular, in the advanced model that we propose here and which includes other parameters, such as internal pressure, gas storage, porosity, and permeability. The principle of inclusion of infused resin and porosity in the balsa core is shown in Figure 5. In this same figure, Figure 5, is represented a μ-tomographic 3D-image reconstruction obtained on a sandwich composite sample (15 s exposure time, pixel size 12.6 μm, 721 images; XRADIA instrument, Oberkochen, Germany). It is very easily observed that the balsa core contains a significant amount of infused resin (in red). When combustion takes place, the balsa core temperature increases, and the resin degrades causing variations of the physical and thermal parameters values, especially porosity and internal pressure, which play a key role in the thermal behavior and material properties. Taking into account a significant amount of resin in the digital model then makes sense for an adequate and more realistic prediction. Thanks to the μ-tomographic 3D image reconstruction, the volume fraction of polyester resin diffused inside the balsa core is considered to be around 5%. Initial porosity of the virgin balsa piece is estimated around 30% in volume fraction [42], while initial porosity of the balsa into the sandwich sample is lightly lower, around 25% in volume fraction. This 5% amount of resin obeys the same thermal behavior as for skins polyester resin. Therefore, equal thermal parameters were used. Of course, it is possible according to our model to choose a resin different from that constituting the skins of the sandwich material. The degradation kinetics of this additional resin is modeled in a manner analogous to the other constituents, in particular, concerning the kinetics law of thermal degradation.

**Table 1:** Material properties for E-glass/polyester composite laminate and balsa core.

Property	Values		References	
	Glass/Polyester	Balsa	Glass/Polyester	Balsa
Fraction volume of fibers (-) V_f_	0.21	-	[43]	
Kinetics rate constant (1/s) A	1000	1.0 × 10^7^	[43]	[33]
Activation energy (J/mol) E_a_	50,000	116,488	[43]	[33]
Reaction order (-) n	1	1	[43]	[33]
Remaining Mass Fraction (-) α	0.01	0.01	[43]	[33]
Heat of decomposition (J/kg) Q_p_	−234,460	556,000	[43]	[33]
Density of balsa core (kg/m^3^) ρ_balsa_	-	126		[29]
Density of glass fiber (kg/m^3^] ρ_fiber_	2694	-	[43]	
Density of polyester (kg/m^3^) ρ_matrix_	1102	-	[43]	
Thermal conductivity of balsa core (W/(m K)) k_balsa_	-	0.2		[33]
Thermal conductivity of glass fiber (W/(m K)) k_fiber_	1.04	-	[43]	
Thermal conductivity of polyester (W/(m K)] k_matrix_	0.19	-	[43]	
Specific heat of initial/final balsa core (J/(kg K)) c_p,balsa_	-	1420/3194		[33]
Specific heat of glass fiber (J/(kg K)) c_p,fiber_	760	-	[43]	
Specific heat of polyester (J/(kg K)) c_p,matrix_	1600	-	[43]	
Specific heat of gas for balsa core (J/(kg K)) c_pg,balsa_	-	1009		[33]
Specific heat of gas for polyester (J/(kg K)) c_pg,matrix_	2386.5	-	[44]	
Thickness of the skin (front/back) and balsa core (m) L	2.5/2.0 × 10^−3^	14 × 10^−3^		
Virgin coefficient of linear thermal expansion (1/K) α_v_	9 × 10^−6^	3 × 10^−5^	[45]	[42]
Char coefficient of linear thermal expansion (1/K) α_c_	1.1 × 10^−5^	0	[45]	[42]
Virgin material permeability (m^2^) γ_v_	3.19 × 10^−16^	9.0 × 10^−12^	[46]	[46]
Char material permeability (m^2^) γ_c_	1.00 × 10^−10^	8.7 × 10^−12^	[46]	[46]
Molecular weight of gases (kg/mol) M	18.35 × 10^−3^	18.35 × 10^−3^	[18]	[46]
Room temperature (°C) T_∞_	20	-		
Room pressure (Pa) P	101,325	-		
Pressure on the back surface(Pa) P_bs_	101,325	-		

**Figure 6:**
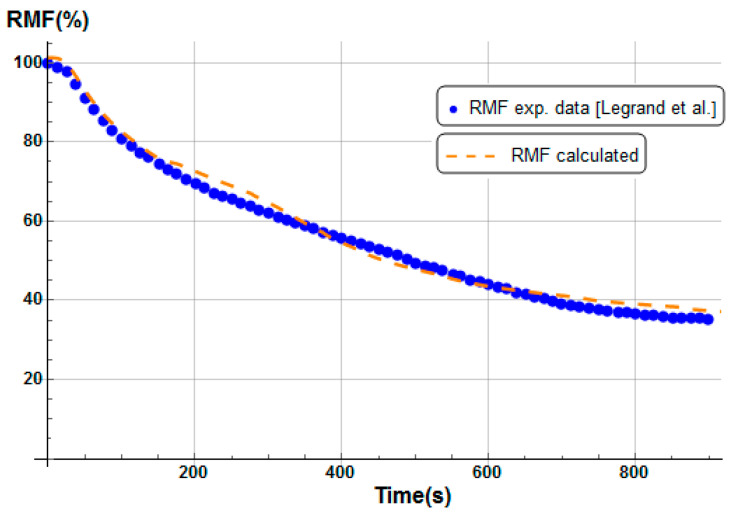
Remaining mass fraction (RMF) profile. Comparison between numerical prediction (this work) as a function of time and the experimental data from Legrand et al. [29] for an E-glass/polyester/balsa sandwich composite at a heat flux of 50 kW/m^2^.

Numerical results were obtained for a heat flux of 50 kW/m^2^. Figure 6 shows the total mass loss of the E-glass/polyester/balsa sandwich sample, following the conversion of unit control volumes to real volumes shown in Figure 2, and using Equation (13) to calculate the remaining mass fraction of the sandwich sample. Contributions of each constituent of the sandwich material (front skin, core, and back skin) were calculated as a numerical average between mass loss profiles as a function of time, through each constituent thickness, using 100 integration points for each one. We included in Figure 6 experimental results measured by Legrand et al. [29] for an appropriate comparison. The experimental data were measured on an ATLAS Cone2 cone calorimeter in the same condition using a radiative source having a heat flux of 50 kW/m^2^. Both numerical and experimental curves show excellent agreement.

**Figure 7:**
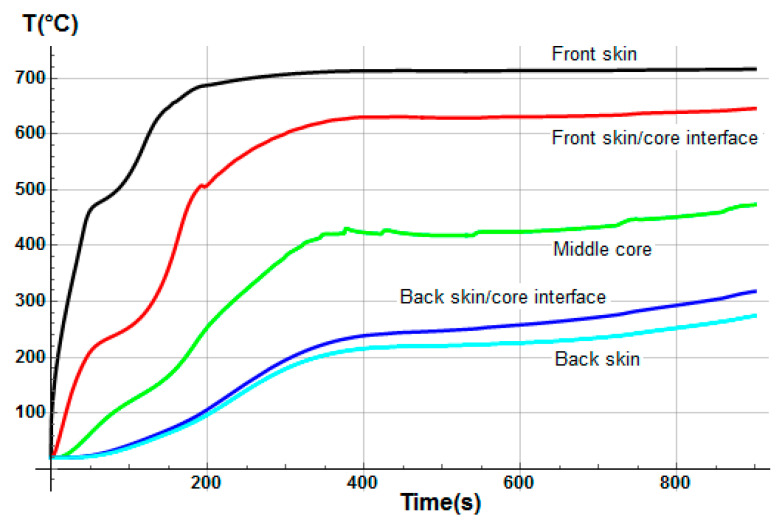
Temperature profile for an E glass/polyester/balsa sandwich composite at a heat flux of 50 kW/m^2^.

Temperature profiles through sandwich thickness were also determined numerically (Figure 7). The model calculates the instantaneous temperature T(x,t) for each *x*-coordinate in the thickness direction of the sandwich, which of course evolves differently depending on the thermal properties of each constituent of the sandwich sample. Thus, the global mass loss is a consequence of the progressive mass loss of each component. It clearly highlights the delay in term of degradation kinetics as a function of the sandwich material through-thickness. First, the mass loss is due to the decomposition of the front skin, in which the upper surface reaches rapidly a temperature around 350 °C, allowing the polyester matrix pyrolysis reaction to start. This decomposition continues up to a temperature of approximately 475 °C, when the polyester matrix becomes completely decomposed in char material. Just before the end of the complete matrix decomposition of the front skin, temperature at the core upper surface reaches a value of around 250 °C, high enough to start the decomposition reaction of balsa organic structures, in particular, the hemicellulose, then the cellulose thermal degradations [47], which progresses up to about 450 °C. This balsa decomposition reaction takes into account the amount of resin inside it, in which the decomposition kinetics are similar to the resin of the skin’s matrix. In parallel with the decomposition process of balsa, the mass loss of the back skin starts as a consequence of the thermal inertia and of the thermal and transport properties of the core made in balsa. The back skin reaches, after 800 s, a high enough temperature to also produce a complete decomposition of its polyester matrix, following a thermal degradation behavior similar to the one described in the case of the front skin. Although the progression of the back skin decomposition is evidently slower, due to the transient and non-linear nature of the heat and decompositions equations involved in the thermal model, leading to different thermal decomposition gradients for each constituent of the sandwich sample.

The mass loss kinetics prediction of each constituent of the sandwich sample for a heat flux of 50 kW/m^2^ is in agreement with typical TGA curves observed in the literature [43,47,48]. Moreover, the sandwich total mass loss (Figure 6) is in very good agreement with measured values [29]. These results demonstrate that the present thermal model for sandwich composite materials allows for correctly predicting the temperature and the mass loss at a heat flux of 50 kW/m^2^.

#### 3.1.2. Glass/Vinyl Ester/Balsa Sandwich Composite

In order to ensure the robustness of the present thermal model, a second type of sandwich composite was used. The thermal behavior of sandwich composite specimens was simulated, in which the skins are made of 5 mm thickness laminate of vinyl ester resin (Derakane 411-350, without flame retardant fillers or additives; Ashland Composite Polymers) and plain-woven E-glass fabric (800 g/m^2^, (0/90) stacking sequence; Owen Corning). The fraction volume of the fibers is 55%. The core consists of a 30 mm thickness balsa wood, embraced between 5 mm skins, in order to obtain a 40 mm thickness symmetric sandwich sample, similar to the one used by Feih et al. [34]. Table 2 summarizes the material properties.

**Figure 8:**
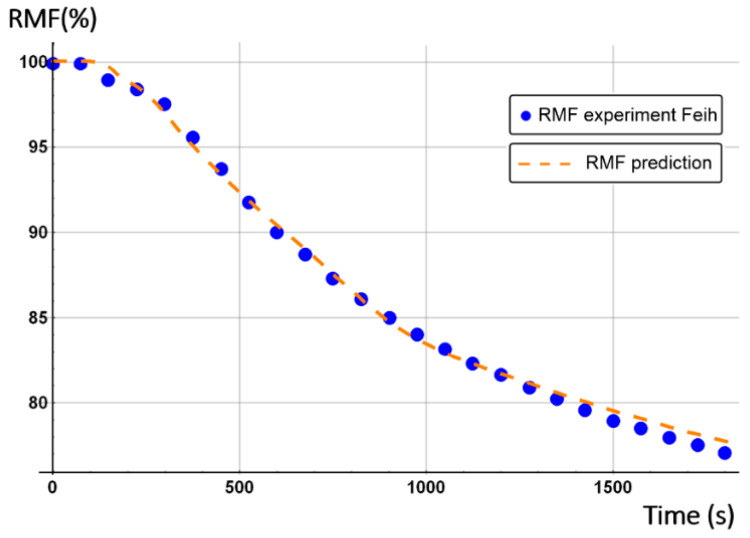
Remaining mass fraction (RMF) profile. Comparison between numerical prediction (this work) as a function of time and the experimental data from Feih et al. [34] for an E-glass/vinyl ester/balsa sandwich composite at a heat flux of 50 kW/m^2^.

**Figure 9:**
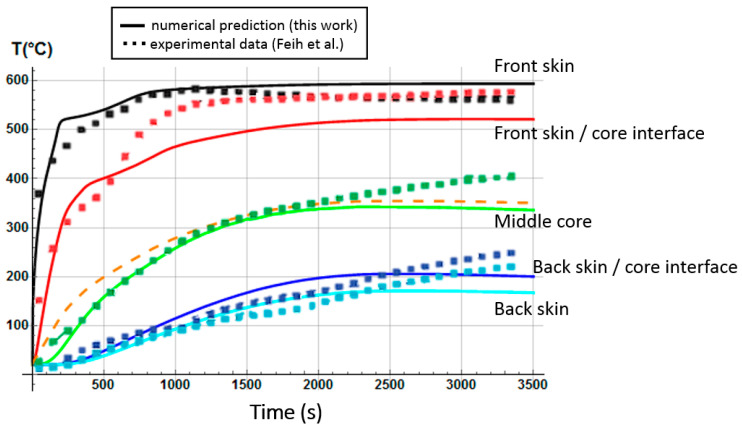
Temperature profiles. Comparison between our numerical prediction as a function of time (straight lines; average numerical temperature in orange dashed line) and the experimental data (dot points) from Feih et al. [34] for an E-glass/vinyl ester/balsa sandwich composite at a heat flux of 50 kW/m^2^.

The initial porosity of balsa into the present sandwich sample is estimated around 20% in volume fraction. Thus, we considered a vinyl ester resin volume fraction of 10% diffused inside the balsa core, as a virgin balsa piece has an initial porosity around 30% in volume fraction [42]. Numerical results were obtained for a heat flux of 50 kW/m^2^. Figure 8 shows the time dependence of the remaining mass fraction (RMF) of the glass/vinyl ester/balsa sandwich sample, taking into account contributions of each sandwich constituent (front skin, core, and back skin). We included in Figure 8 the experimental data measured by Feih et al. [34] in order to compare and validate again the present numerical model. One could observe that the present thermal model makes it possible to faithfully reproduce the mass loss of the material during a fire test. Then, it can be used for predicting the temperature profiles as a function of time, as shown in Figure 9. Temperature is predicted through the sandwich thickness, at five representative surfaces of the sample (upper surface of front skin x = 0 mm, lower surface of front skin x = 5 mm, middle surface of core x = 20 mm, upper surface of back skin x = 35 mm, and lower surface of back skin x = 40 mm). Figure 9 also includes the experimental measurements of Feih et al. [34], for comparison.

We observe in Figure 8 that the global mass loss follows a classical Arrhenius curve, in relation with the thermal degradation description of each constituent through Equation (5). Comparing with Figure 9, one could notice that the mass loss begins when the front skin reaches a temperature around 400 °C, in relation with the vinyl ester matrix temperature decomposition also evaluated around 400 °C, as it can be verified from typical TGA curves [34,47,48]. The thermal equilibrium is reached after about 1000 s for the front skin and 2500 s for the back skin. Exposed to a constant heat flux of 50 kW/m^2^, the material exhibits a large thermal gradient between the front and back surfaces of 400 °C due to the low thermal conductivity of the balsa core and its initial thickness of 30 mm. For a sufficiently long exposure time, the upper front surface of the front skin reaches a temperature of about 600 °C, while the lower surface of the back skin exhibits a temperature of 180 °C. The temperature gradient implies also a gradient in the composite sandwich material with non-linear degradation, depending on the through-thickness x-coordinate and of the exposure time. It can be noted in Figure 9 that the simulated curves and the experimental curves do not overlap perfectly. Indeed, our model, even including a large number of parameters and material properties, assumes that volatile flow is one-dimensional in the through-thickness direction and that no damage, such as cracks or debonding, appears during the fire test. These mechanical phenomena could be relatively important on interfaces between sandwich components, as observed in Figure 9, because of their influence on heat transfers [34]. In reality, volatile gases flow also in the longitudinal direction of the sample (from and to the edges). Moreover, as the temperature increases, the internal pressure (due to gas and to mechanical strain) also increases, creating cracks and debonding, which induces slowing or accelerations on the temperature variation. In addition, during the pyrolysis of the sample, often analyzed on a cone calorimeter, commonly observed is a delocalization of the thermocouples then inducing a higher value of temperature for the one located at the upper skin/core interface, as well as for all the thermocouple for long measurement times. Based on these considerations, our thermal model well allows to predict the temperature variations in any point of the material, despite the small variations observed.

Temperature profiles produce a global mass loss of about 20% (Figure 8) of the initial sandwich sample mass, due to an initial decomposition of the front skin matrix, followed by a progressive decomposition of the balsa core organics fibers [47] composed of hemicellulose and cellulose. However, back skin does not decompose, as it does not reach a high enough temperature to start the vinyl ester matrix degradation. That can be numerically observed, thanks to the mass loss kinetics for each constituent, which can be analyzed in more detail, thanks to the remaining mass fraction for any through-thickness x-coordinate. Moreover, balsa core presents two different mass loss kinetics corresponding to the balsa organics fibers and to the amount of resin inside balsa core, both having different thermal kinetics parameters.

Even if some parameters and thermal processes are not yet included in our model (one-dimensional model, no damage, such as cracks or debonding, appears), the numerical predictions for E-glass/vinyl ester/balsa sandwich composite materials, subject to a heat flux of 50 kW/m^2^, are in very good agreement with literature results, showing the robustness of our model lies in the fact that non-typical phenomena evolutions are also available.

### 3.2. Mechanical Properties Prediction

In order to estimate the mechanical properties of sandwich composite materials, temperature and mass loss profiles obtained from the thermal model are used to calculate post-combustion mechanical properties through the equivalent flexural modulus expressed by Equation (24). For that, the combustion advancing front is calculated for each constituent of the sandwich sample, since each constituent has its own rate of degradation α(x,t), depending on its nature and geometry, and also its position within the sample with respect to the incident thermal flux. The equivalent flexural modulus can be deduced from the sum of all of the constituent contributions.

#### 3.2.1. Glass/Polyester/Balsa Sandwich Composite

**Figure 10:**
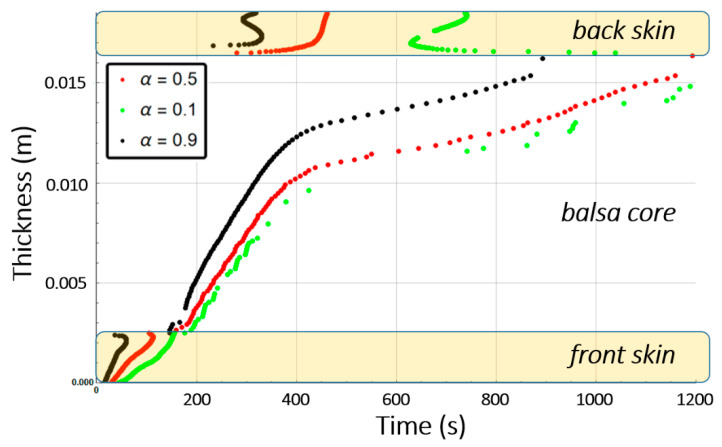
Numerical simulation prediction of the combustion advancing front for an E-glass/polyester/balsa sandwich composite material at a heat flux of 50 kW/m^2^: three degradation levels α(x,t) are represented.

Figure 10 shows the combustion advancing front for three different degradation rates: 50% of degradation (α(x,t)=0.5, intermediate decomposition), 90% of degradation (α(x,t)=0.1, almost total decomposition), and 10% of degradation (α(x,t)=0.9, low decomposition) for each one of the sandwich specimen constituent (skins and core) at a heat flux of 50 kW/m^2^. We remark that α(x,t) represents the remaining virgin material during thermal degradation. Thus, it is used here as an indicator to define the position of the advancing front in the through thickness x-coordinate, assuming a fixed decomposition level to estimate the front, as explained in Section 2.2.

**Figure 11:**
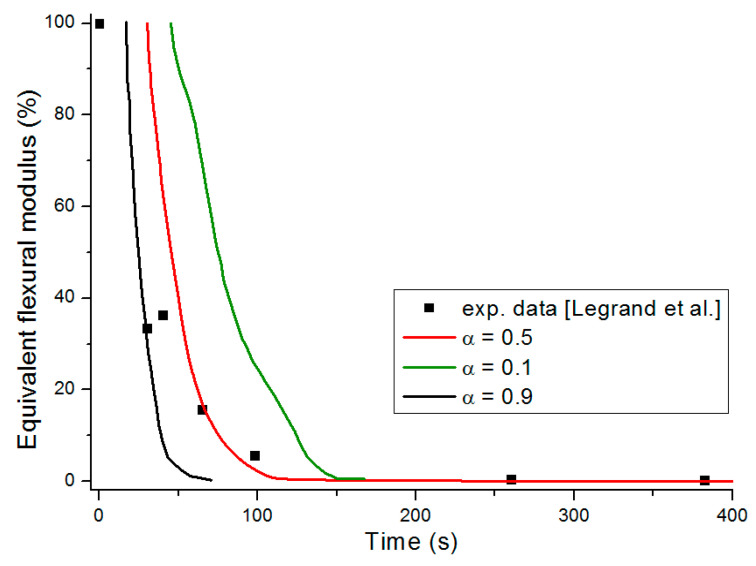
Apparent equivalent flexural modulus (contribution of only the front skin). Numerical simulation predictions as a function of time for an E-glass/polyester/balsa sandwich composite material at a heat flux of 50 kW/m^2^. Numerical predictions are superposed with the experimental data (█) measured by Legrand et al. [29].

The equivalent flexural modulus (Figure 11) was calculated considering only the mechanical properties of the front skin because failure of a sandwich composite is assumed to occur by unstable microbucking (kinking) of the front skin, leading to catastrophic failure of the whole sandwich. That is experimentally observed, thanks to three point bending flexural tests [29] showing degradation time dependence of the flexural modulus. The front skin is submitted to compression stress and has the highest degradation rate, which quickly yields its failure. Then, the balsa core and back skin are assumed to suddenly collapse as they are not capable to withstand the bending state initially experienced by the whole sandwich structure. The strength value of the core is assumed to be well below the skins one, so its contribution can be neglected. Thus, as the front skin degradation progresses and finally collapses, the back skin must withstand practically all the flexion load, which produces also its sudden failure.

**Figure 12:**
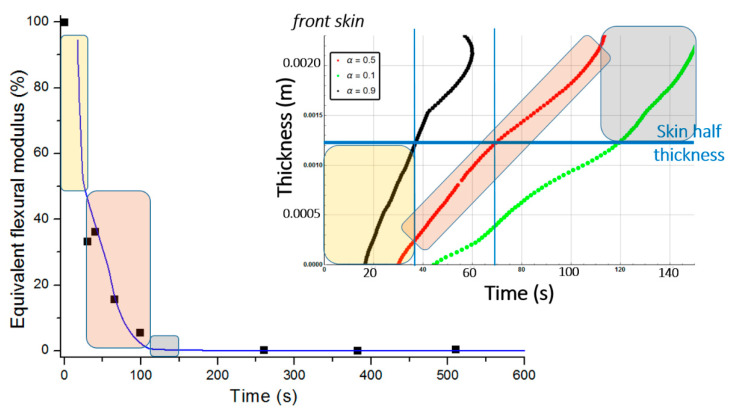
Apparent equivalent flexural modulus (contribution of only the front skin). Numerical predictions, considering a degradation rate gradient (see text for more details) as a function of time for an E-glass/polyester/balsa sandwich composite material at a heat flux of 50 kW/m^2^. Numerical predictions are superposed with the experimental data (█) measured by Legrand et al. [29]. Inserted figure shows the prediction of the combustion advancing front in the front skin for three degradation levels α(x,t).

Figure 11 presents the equivalent flexural modulus only considering the contribution of the front skin supposing the three different degradation levels of α(x,t)=0.5 (intermediate decomposition), 0.1 (almost total decomposition) and 0.9 (low decomposition) for each constituent of the sandwich specimen. Experimental data measured by Legrand et al. [29] are also reported for appropriate comparison. It well shows that the decrease of the flexural modulus is directly linked with the fire exposure time, which modifies the advancing combustion front position and consequently the length of the carbonized layer. For this reason, the experimental data are not completely in agreement with only one of the numerical simulations but are distributed over several curves as a function of the combustion time. This distribution would be equivalent to consider a more sophisticated mechanical model than the current two-layer approach, as presented in Section 2.2, assuming that the contribution of transition layer between virgin and char material to mechanical performances is not negligible. The results can be interpreted on the basis of an almost immediate drop in flexural modulus from the first moment of thermal degradation at a heat flux of 50 kW/m^2^. Thus for a thermal degradation reaching quickly (α(x,t)=0.9) through the thickness of the front skin, the material loses a great part of its mechanical integrity causing, in the first 35 s, a 50% reduction of the flexural modulus. The combustion advancing front propagating progressively, and the temperature increasing, the level of degradation seems to increase and be closer to α(x,t)=0.5 in the whole of the upper skin. Figure 11 shows that the simulated curve for this degradation level fits perfectly with the experimental data for times greater than 60 s. It could be assumed sudden and unstable failures by microbuckling of the front skin inducing a fall of the module at 50% of the maximum strength. The material then keeps a roughly constant level of mechanical properties until the front skin is partially degraded assuming intermediate or high decomposition level (α(x,t)=0.5 or less), causing the flexural modulus to drop to zero. In this sense, a new simulation was undertaken to take into account increasing degradation levels with exposition time. Figure 12 shows the variation of the flexural modulus by considering, for the front skin, a value of α(x,t)=0.9 up to 30 s, α(x,t)=0.1 for times greater than 115 s, and α(x,t)=0.5 for intermediate degradation time. It is interesting to note that these times chosen in correspondence with the degradation levels form remarkable areas that can be connected with the half thickness of the front skin, as shown in the insert of Figure 12. To be more precise in the prediction, especially for first steps of mechanical degradation before any or neglected thermal degradation, other approach should be formulated in order to consider the effect of the temperature on the ply mechanical behavior, and also on a progressive ply by ply failure, by stable plastic kinking, what implies complicated model development to consider local effects of progressive damage at the ply scale. Further experimental analysis, such as microscopic observation for reduced/intermediate mechanical degradation (from ~0% to ~50% of decreasing of flexural modulus) samples presenting any or neglected thermal degradation, are necessary to get useful information concerning first sources of mechanical degradation. However, the results are very encouraging and the agreement with experimental data highly allows the validation of the present approach to predict post-combustion mechanical properties of sandwich composite material with glass/polymer laminated skins and combustible core.

#### 3.2.2. Glass/Vinyl Ester/Balsa Sandwich Composite

**Figure 13:**
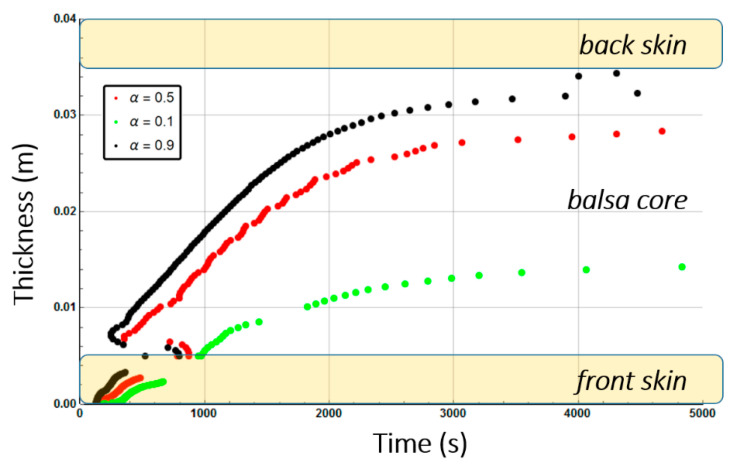
Numerical prediction of the combustion advancing front for an E glass/vinyl ester/balsa sandwich composite material at a heat flux of 50 kW/m^2^: three degradation levels α(x,t) are represented.

Figure 13 shows the combustion advancing front for three different degradation levels: α(x,t)=0.5 (intermediate decomposition), α(x,t)=0.1 (almost total decomposition), and α(x,t)=0.9 (low decomposition) for each one of the Glass/Vinyl ester/balsa sandwich specimen constituent (skins and core) at a heat flux of 50 kW/m^2^. In relation with the observations and discussions made from Figure 8 and Figure 9, Figure 13 clearly shows that the temperature reached for the back skin is not high enough to allow the thermal decomposition thereof. Similarly, for the core, we observe that the balsa degradation is very gradual. Indeed, after a time of 3000 s, a 50% degradation of almost the entire balsa core is noted, as well as that the first tier close to the heat source is degraded to more than 90%.

**Figure 14:**
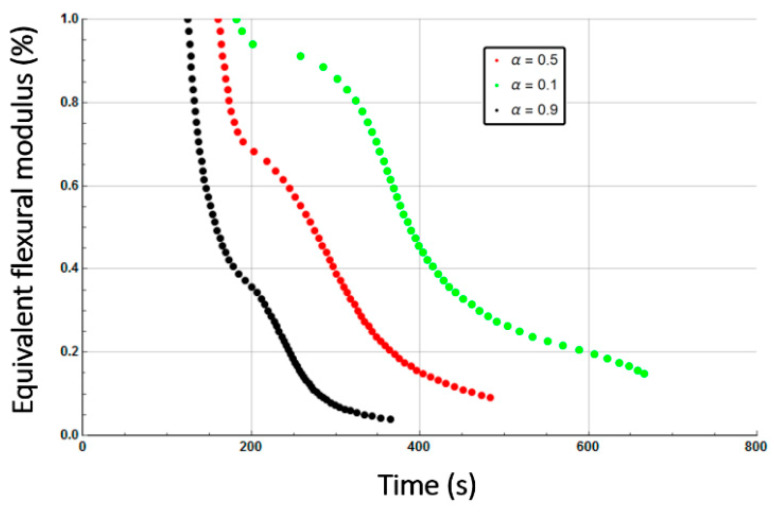
Apparent equivalent flexural modulus (contribution of only the front skin). Numerical predictions as a function of time for an E-glass/vinyl ester/balsa sandwich composite material at a heat flux of 50 kW/m^2^.

**Figure 15:**
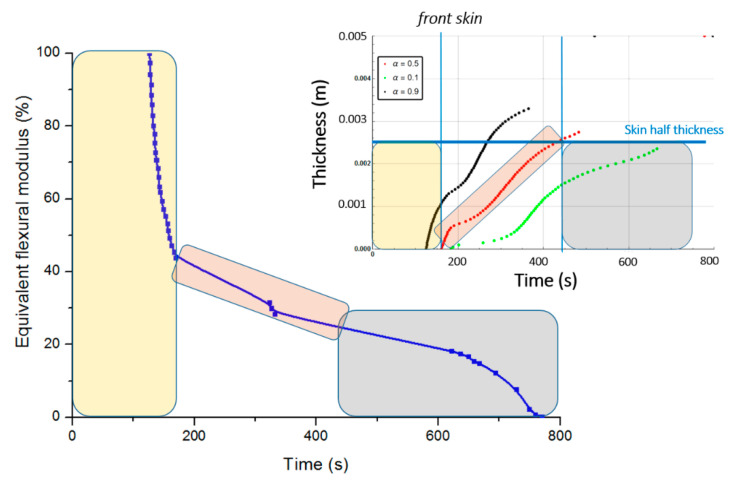
Apparent equivalent flexural modulus (contribution of only the front skin). Numerical predictions, considering a degradation rate gradient (see text for more details) as a function of time for an E-glass/vinyl ester/balsa sandwich composite material at a heat flux of 50 kW/m^2^. Inserted figure shows the prediction of the combustion advancing front in the front skin for three degradation levels α(x,t).

Figure 14 represents the equivalent flexural modulus determined from the mechanical properties of the front skin, and plotted for the three different degradation levels: α(x,t)=0.5, α(x,t)=0.1, and α(x,t)=0.9. Experimentally, the post-combustion flexural modulus is studied performing three point bending flexural tests made on thermal aged samples at different combustion time. During a mechanical stress in flexion, the front skin is submitted to compression stress and has the highest degradation level that quickly produces its failure, as previously explained for an E-glass/polyester/balsa sandwich. Cracking then propagates through the material. The other constituents of the structure should then withstand the overall applied load, which induces a rapid and abrupt rupture of the core and the back skin. As the balsa core strength value is very weak compared to that of the skin, it can be neglected as a first approximation. Thus, one could observe from Figure 14 that the equivalent flexural modulus tends rapidly to zero with failure times between 400 s and 800 s for degradation levels corresponding from α(x,t)=0.9 to α(x,t)=0.1, respectively. The results can be interpreted on the basis of an almost immediate drop in flexural modulus from the first instant of thermal degradation at a heat flux of 50 kW/m^2^, i.e., when at least 20% of the thickness of the front skin is partially degraded to less than 10% (that means assuming α(x,t)≥0.9) after only 200 s of exposure time. Figure 14 shows that the combustion advancing front propagating progressively, in relation with the temperature inside the front skin being between 400 and 500 °C in this constituent (Figure 9). The combustion advancing front then propagates progressively, and the degradation level increases, reaching about 50% (α(x,t)=0.5). Finally, the sandwich composite retains a roughly constant level of mechanical properties during the interval time 400–600 s until the front skin is degraded to more than 50%, corresponding to α(x,t)≤0.1, causing suddenly the flexural modulus to drop to zero. In order to have a representative description of all the degradation of the material, inducing the fall of the flexural modulus, it is therefore necessary to take into account, in reality, the decomposition gradient that it undergoes as a function of time. Figure 15 shows the evolution of the equivalent flexural modulus by taking into account the degradation level gradient through the thickness of the front skin. The mechanical property drop of the material then becomes more representative and conforms to reality.

## 4. Hygro-Thermal Durability in Fire Condition

### 4.1. Hygro-Thermal Model Development

**Figure 16:**
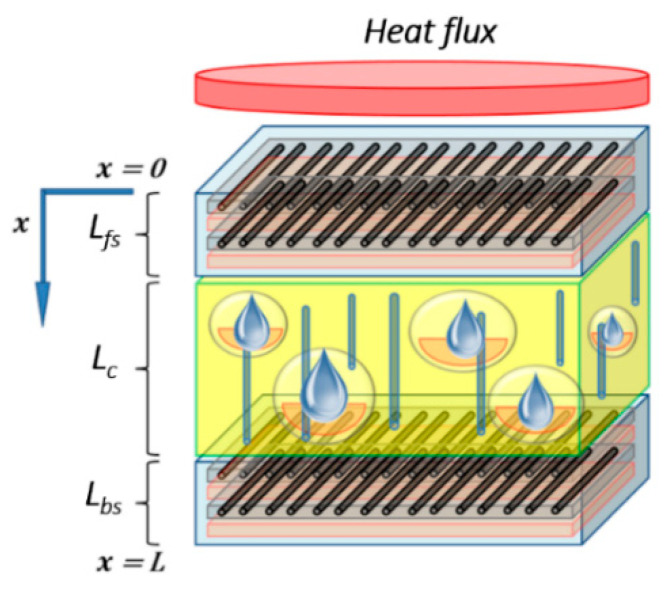
Scheme of the hygro-thermal model for sandwich composite material exposed to a constant unidirectional heat flux. The model considers the presence of (i) porosities and moisture absorption inside the skins, and of (ii) porosities, resin diffusion and moisture absorption inside the balsa core.

Water content can be even more influent on the thermal behavior of sandwich materials, especially if they are constituted with porous core as balsa wood. Moisture absorption can drastically modify the global properties and material durability [49,50,51,52]. In this context, assumptions were made to model the one-dimensional hygro-thermal response of sandwich composites with polymer laminated skins and combustible core. To model the thermal behavior of a material with initial water content in core and skins, we proceed in a similar way as proposed in the model of Legrand et al. for laminated materials [21]. This initial water is mostly stored inside skins and core porosities, and to a lesser extent inside skins matrix resin and core organic fibers, as shown in Figure 16. Two steps are considered: (i) free water desorption is considered (stored in porosities, without Van der Waals or hydrogen bonds with the molecular network); and (ii) bounded water is considered (with weak bonds) ejected at higher temperatures.

Water desorption was modeled using Equation (25), representing an additional equation, based on the dehydration equation proposed by Sand et al. [53], to include in the PDEs system to solve:

$$\frac{\partial m_{H_{2}O,fs}\left(x,t\right)}{\partial t}=-\frac{\partial }{\partial t}\left(\frac{m_{composite,fs}\left(x,t\right)\;c_{p,fs}\left(x,t\right)\left[T\left(x,t\right)-T_{sat,fs}\right]}{\Delta h_{fg,fs}\left(x,t\right)}\right)\;\mathrm{for}\;0\leq x\leq L_{fs},$$

$$\frac{\partial m_{H_{2}O,c}\left(x,t\right)}{\partial t}=-\frac{\partial }{\partial t}\left(\frac{m_{composite,c}\left(x,t\right)\;c_{p,c}\left(x,t\right)\left[T\left(x,t\right)-T_{sat,c}\right]}{\Delta h_{fg,c}\left(x,t\right)}\right)\;\mathrm{for}\;L_{fs}\leq x\leq L_{fs}+L_{c},$$

(25) $$\frac{\partial m_{H_{2}O,bs}\left(x,t\right)}{\partial t}=-\frac{\partial }{\partial t}\left(\frac{m_{composite,bs}\left(x,t\right)\;c_{p,bs}\left(x,t\right)\left[T\left(x,t\right)-T_{sat,bs}\right]}{\Delta h_{fg,bs}\left(x,t\right)}\right)\;\mathrm{for}\;L_{fs}+L_{c}\leq x\leq L_{fs}+L_{c}+L_{bs}.$$

In Equation (25), $m_{H_{2}O,fs,c,bs}\left(x,t\right)$ is the instantaneous water mass contained inside each sandwich constituent material (*fs*, *c*, and *bs* being for front skin, core, and back skin, respectively). $T_{sat,fs,c,bs}$ is the fibers saturation temperature of each sandwich constituent material. In the first step, *T_sat_* = 373.15 K for free water desorption. In the second step, $T_{sat,fs,c,bs}$ is slightly higher for bounded water and depends of the value $X_{fsp}$, which is the mass fraction of water at fiber saturation point. This value is much higher for organic fibers of balsa wood core ($X_{fsp,c}≃0.2)$ than polymer fibers of vinyl ester or polyester ($X_{fsp,fs,bs}≃0.001)$ [53]. $\Delta h_{fg,fs,c,bs}\left(x,t\right)$ is the latent heat of water which depends of the temperature T(x,t) and also of the water mass fraction of each constituent $\left(X_{H_{2}O,fs,c,bs}\left(x,t\right)=\frac{m_{H_{2}O,fs,c,bs}\left(x,t\right)}{m_{composite,fs,c,bs}\left(x,t\right)}\right)$ for the bounded water desorption step. This later takes different expressions for free liquid and bounded water molecules, as proposed in Equations (26)–(29).

(26) $$\Delta h_{l,fs,c,bs}\left(x,t\right)\left[J/\mathrm{kg}\right]=1000\left[3179-2.5\;T\left(x,t\right)\right],$$

(27) $$\Delta h_{desorp,fs,c,bs}\left(x,t\right)\left[J/\mathrm{kg}\right]=1000\left[1176.2e^{-15X_{H_{2}O,fs,c,bs}\left(x,t\right)}\right],$$

(28) $$\Delta h_{fg,fs,c,bs}\left(x,t\right)=\Delta h_{l,fs,c,bs}\left(x,t\right)\;\mathrm{for}\;X_{H_{2}O,fs,c,bs}\left(x,t\right)\geq X_{fsp,fs,c,bs},$$

(29) $$\Delta h_{fg,fs,c,bs}\left(x,t\right)=\Delta h_{l,fs,c,bs}\left(x,t\right)+\Delta h_{desorp,fs,c,bs}\left(x,t\right)\;\mathrm{for}\;X_{H_{2}O,fs,c,bs}\left(x,t\right)<X_{fsp,fs,c,bs}.$$

Heat equation is modified as follows so that it explicitly considers the water content of each constituent (front skin, core, and back skin):

$$\begin{array}{c} \left[m_{composite,fs}\left(x,t\right)\left(c_{p,fs}\left(x,t\right)+c_{{pvH}_{2}O,fs}\left(x,t\right)\right)+m_{g,fs}\left(x,t\right)c_{pg,fs}\left(x,t\right)\right]\frac{\partial T\left(x,t\right)}{\partial t}=\\ \frac{\partial }{\partial x}\left\{\left[k_{g,fs}\left(x,t\right)\phi_{fs}\left(x,t\right)+k_{x,fs}\left(x,t\right)\left(1-\phi_{fs}\left(x,t\right)\right)\right]\frac{\partial T\left(x,t\right)}{\partial x}\right\}{\Delta x}_{fs}\left(x,t\right){\Delta A}_{fs}-\\ {\dot{m}}_{gtotal,fs}\left(x,t\right)c_{pg,fs}\left(x,t\right)\frac{\partial T\left(x,t\right)}{\partial x}{\Delta x}_{fs}\left(x,t\right)-\frac{\partial m_{composite,fs}\left(x,t\right)}{\partial t}[Q_{p,fs}+h_{s,fs}\left(x,t\right)-\\ h_{g,fs}\left(x,t\right)-h_{{vH}_{2}O,fs}\left(x,t\right)]\;\mathrm{for}\;0\leq x\leq L_{fs}, \end{array}$$

$$\begin{array}{c} \left[m_{composite,c}\left(x,t\right)\left(c_{p,c}\left(x,t\right)+c_{{pvH}_{2}O,c}\left(x,t\right)\right)+m_{g,c}\left(x,t\right)c_{pg,c}\left(x,t\right)\right]\frac{\partial T\left(x,t\right)}{\partial t}=\\ \frac{\partial }{\partial x}\{[k_{g,c}\left(x,t\right)\phi_{c}\left(x,t\right)+k_{x,c}\left(x,t\right)\left(1-\phi_{c}\left(x,t\right)\right)]\frac{\partial T\left(x,t\right)}{\partial x}\}{\Delta x}_{c}\left(x,t\right){\Delta A}_{c}-\\ {\dot{m}}_{gtotal,c}\left(x,t\right)c_{pg,c}\left(x,t\right)\frac{\partial T\left(x,t\right)}{\partial x}{\Delta x}_{c}\left(x,t\right)-\frac{\partial m_{composite,c}\left(x,t\right)}{\partial t}[Q_{p,c}+h_{s,c}\left(x,t\right)-h_{g,c}\left(x,t\right)-\\ h_{{vH}_{2}O,c}\left(x,t\right)]\;\mathrm{for}\;L_{fs}\leq x\leq L_{fs}+L_{c}, \end{array}$$

(30) $$\begin{array}{c} \left[m_{composite,bs}\left(x,t\right)\left(c_{p,bs}\left(x,t\right)+c_{{pvH}_{2}O,bs}\left(x,t\right)\right)+m_{g,bs}\left(x,t\right)c_{pg,bs}\left(x,t\right)\right]\frac{\partial T\left(x,t\right)}{\partial t}=\\ \frac{\partial }{\partial x}\left\{\left[k_{g,bs}\left(x,t\right)\phi_{bs}\left(x,t\right)+k_{x,bs}\left(x,t\right)\left(1-\phi_{bs}\left(x,t\right)\right)\right]\frac{\partial T\left(x,t\right)}{\partial x}\right\}{\Delta x}_{bs}\left(x,t\right){\Delta A}_{bs}-\\ {\dot{m}}_{gtotal,bs}\left(x,t\right)c_{pg,bs}\left(x,t\right)\frac{\partial T\left(x,t\right)}{\partial x}{\Delta x}_{bs}\left(x,t\right)-\frac{\partial m_{composite,bs}\left(x,t\right)}{\partial t}[Q_{p,bs}+h_{s,bs}\left(x,t\right)-\\ h_{g,bs}\left(x,t\right)-h_{{vH}_{2}O,bs}\left(x,t\right)]\;\mathrm{for}\;L_{fs}+L_{c}\leq x\leq L_{fs}+L_{c}+L_{bs}, \end{array}$$

with

$$m_{composite,fs,bs}\left(x,t\right)=m_{m,fs,bs}\left(x,t\right)+m_{fiber,fs,bs}+m_{H_{2}O,fs,bs}\left(x,t\right),$$

(31) $$m_{composite,c}\left(x,t\right)=m_{m,c}\left(x,t\right)+m_{H_{2}O,c}\left(x,t\right),$$

and

(32) $${\dot{m}}_{gtotal,fs,c,bs}\left(x,t\right)={\dot{m}}_{g,fs,c,bs}\left(x,t\right)+{\dot{m}}_{vH_{2}O,fs,c,bs}\left(x,t\right),$$

and

(33) $$\begin{array}{c} \frac{\partial m_{composite,fs,c,bs}\left(x,t\right)}{\partial t}=m_{composite,fs,c,bs}\left(x,t\right)\;A_{fs,c,bs}\;{\left[\alpha_{fs,c,bs}\left(x,t\right)\right]}^{n_{fs,c,bs}}e^{-\frac{E_{a,fs,c,bs}}{R\;T\left(x,t\right)}}\\ \mathrm{for}\;m_{H_{2}O,fs,c,bs}\left(x,t\right)=0. \end{array}$$

The specific heat (Equation (34)) of each constituent is modified as follows, in a similar way as in Reference [21], to take into account the influence of water content:

(34) $$c_{pvH_{2}O,fs,c,bs}\left(x,t\right)=\frac{f_{c}\;\Delta h_{fg,fs,c,bs}\left(x,t\right)\;X_{H_{2}O,fs,c,bs}\left(x,t\right)}{T\left(x,t\right)-T_{sat,fs,c,bs}}.$$

In Equation (34), $f_{c}$ is a correction factor to take into account an additional energy due to the dehydration process $\left(f_{c}≃1.33\right)$. Finally, $c_{pH_{2}O,fs,c,bs}\left(x,t\right)$ allows to calculate an additional contribution to the enthalpy $h_{vH_{2}O,fs,c,bs}\left(x,t\right)$, produced by each constituent desorption, which must be included in Equation (30) to ensure the energy conservation.

The mass conservation Equation (8) must also be adapted in order to consider the water mass contribution. That allows to distinguish the calculation of volatiles mass storage and evaporation pressure, and of matrix and balsa decomposition processes, for skins and core, respectively. This assumption allows to evaluate individually the contribution of each process for each constituent of the sandwich composite, as shown in Equations (35)–(37):

(35) $$\begin{array}{c} -\frac{\partial m_{composite,fs,c,bs}\left(x,t\right)}{\partial t}=\frac{\partial m_{g,fs,c,bs}\left(x,t\right)}{\partial t}+\frac{\partial {\dot{m}}_{g,fs,c,bs}\left(x,t\right)}{\partial t}\Delta x_{fs,c,bs}\left(x,t\right)+\frac{\partial m_{vH_{2}O,fs,c,bs}\left(x,t\right)}{\partial t}+\\ \frac{\partial {\dot{m}}_{vH_{2}O,fs,c,bs}\left(x,t\right)}{\partial t}\Delta x_{fs,c,bs}\left(x,t\right), \end{array}$$

(36) $$-\frac{\partial \left(m_{m,fs,c,bs}\left(x,t\right)\right)}{\partial t}=\frac{\partial m_{g,fs,c,bs}\left(x,t\right)}{\partial t}+\frac{\partial {\dot{m}}_{g,fs,c,bs}\left(x,t\right)}{\partial t}\Delta x_{fs,c,bs}\left(x,t\right),$$

(37) $$-\frac{\partial m_{H_{2}O,fs,c,bs}\left(x,t\right)}{\partial t}=\frac{\partial m_{vH_{2}O,fs,c,bs}\left(x,t\right)}{\partial t}+\frac{\partial {\dot{m}}_{vH_{2}O,fs,c,bs}\left(x,t\right)}{\partial t}\Delta x_{fs,c,bs}\left(x,t\right),$$

with the subscripts g related to the gases ejected by skins or core decomposition, and $vH_{2}O$ referring to water vapor. During the first step of the water desorption process, internal pressure for each constituent is so calculated using Equation (10) up to $X_{fsp,fs,c,bs}$. During the second step, internal pressure is determined using Equation (38) [53], with $P_{vH_{2}O,fs,c,bs}^{sat}$ referring to the saturation pressure when $X_{H_{2}O,fs,c,bs}\left(x,t\right)=X_{fsp,fs,c,bs}$, and being different for each constituent of the sandwich composite.

(38) $$\begin{array}{c} P_{vH_{2}O,fs,c,bs}\left(x,t\right)=P_{vH_{2}O,fs,c,bs}^{sat}\;\left[1-{\left(1-\frac{\;X_{H_{2}O,fs,c,bs}\left(x,t\right)}{X_{fsp,fs,c,bs}}\right)}^{6.453\cdot 10^{-3}\;T\left(x,t\right)}\right]\;\\ \mathrm{for}\;X_{H_{2}O,fs,c,bs}\left(x,t\right)<X_{fsp,fs,c,bs}. \end{array}$$

New initial and boundary conditions involving the new variables related to water content must be included in order to become the hygro-thermal problem in a mathematically resolvable system of PDEs. These new conditions are related to each constituent and are summarized in Equations (39) and (40).

(39) $$\begin{array}{c} m_{H_{2}O,fs,c,bs}\left(x,0\right)=m_{H_{2}O0,fs,c,bs},\;m_{vH_{2}O,fs,c,bs}\left(x,0\right)=m_{vH_{2}O0,fs,c,bs},\;\\ {\dot{m}}_{vH_{2}O,fs,c,bs}\left(x,0\right)=0,\;P_{vH_{2}O,fs,c,bs}\left(x,0\right)=P_{vH_{2}O0,fs,c,bs}\;\mathrm{for}\;0\leq x\leq L_{total}, \end{array}$$

(40) $${\dot{m}}_{vH_{2}O,bs}\left(L_{total},t\right)=0,\;P\left(L_{total},t\right)=P_{s}\;\mathrm{for}\;x=L_{total}\;\mathrm{and}\;\forall t>0.$$

This new formulation of hygro-thermal problem gives access to the temporal and through-thickness evolution of all variables involved. Obviously, temperature and matrix mass loss profiles are influenced by water content. The water mass contributions of each constituent, in relation to the total remaining mass fraction of the whole sandwich composite, must be calculated taking into account the relative thickness of each constituent. Then, Equation (13) must be adapted into Equation (41) as follows:

(41) $$RMF_{sandwich}\left(x,t\right)=\frac{\left(m_{fs}\left(x,t\right)+m_{H_{2}O,fs})\left(x,t\right)\;L_{fs}+(m_{c}\left(x,t\right)+m_{H_{2}O,c})\;L_{c}+(m_{bs}\left(x,t\right)+m_{H_{2}O,bs}\right)\;L_{bs}}{(m_{sandwich0}+m_{H_{2}O0)}\;L_{total}}.$$

Thanks to this hygro-thermal formulation in the case of sandwich composite material, one could predict the post-combustion mechanical properties using the model described in Section 2.2.

### 4.2. Hygro-Thermo-Mechanical Durability

#### 4.2.1. Glass/Polyester/Balsa Sandwich Composite

**Figure 17:**
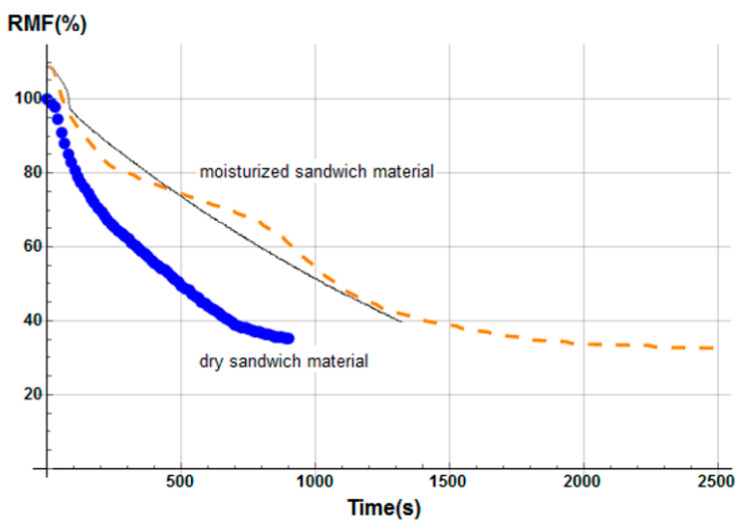
Remaining mass fraction (RMF) profile. Comparison of the numerical predictions as a function of time between thermal (blue dot line) and hygro-thermal (orange dashed line) models. Experimental data from Legrand et al. [29] are added (black line) for the moisturized sandwich material. Compound: E-glass/polyester/balsa sandwich composite at a heat flux of 50 kW/m^2^.

**Figure 18:**
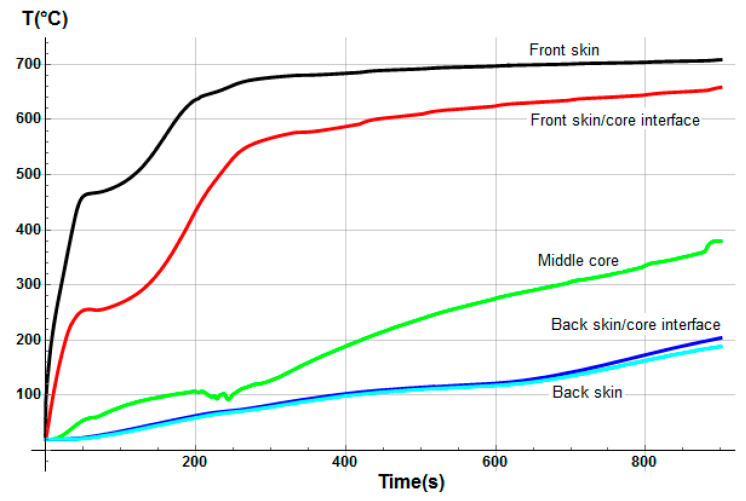
Temperature profile for an E-glass/polyester/balsa sandwich composite with a 30% water content in relation to the porosity volume fraction of the balsa core, at a heat flux of 50 kW/m^2^.

The hygro-thermal model was applied in the case of a heat flux of 50 kW/m^2^ for a glass/polyester/balsa sandwich composite material. Figure 17 presents the time dependence of the total mass loss for the E-glass/polyester/balsa sandwich material, with an initial water mass fraction content of 30% in relation to the porosity volume fraction of the balsa core. The agreement between predicted and experimental results is very good temperature profiles through sandwich thickness were also determined numerically (Figure 18). The temperature of the upper skin follows approximately the same evolution as for the dry material (Figure 7). This is normal because the hydration of the skins has not been considered here. The surface temperature very quickly reaches 475 °C, causing a complete decomposition of the polyester matrix of the upper skin, and only the glass fibers remain. This occurs during the first 50 to 100 s of exposure to the heat flow. During the same period of time, it is observed a sudden decrease in the overall mass of the material of the order of 10%. The surface of the balsa in contact with the upper skin sees its temperature brought to around 250 °C, which is sufficient to cause an onset of thermal degradation of the balsa hemicellulose [47,54]. The mass loss kinetics of the material then changes of slope to follow the one induced by the degradation of the balsa. During this period, the resin infused into the balsa core also pyrolyzes progressively. By observing the temperature variation in the middle of the balsa core, we can see that it is very slow, slowed down by the fact that the balsa is hydrated. Figure 18 shows, in particular, a plateau at 100 °C between 200 and 300 s indicating the moment when the free and bounded water evaporates from the balsa structure. The degradation is slowed up to 800 s of thermal exposure. From 800 s, the average temperature in the balsa reaches about 350 °C. At the same time, there is a change in slope of the kinetics of the overall mass loss of the sandwich material with an acceleration of the degradation. This temperature corresponds exactly to the maximum degradation temperature of cellulose in hydrated balsa as observed by Thermogravimetric Analysis (TGA) measurements [47]. The material degrades sharply and thus loses 30% in weight mass, until the time 1400 s, when the active pyrolysis of balsa is finished, to give rise to passive pyrolysis, which is physically interpreted by a very slow decomposition of lignin. From that moment, the upper skin is completely decomposed, and one could note a systematic shift between the kinetics of the two materials (dry and moisturized) mass loss of about 600 s, such that the hydrated sandwich composite material undergoing a much slower thermal degradation. Water content works as a thermal barrier giving an isolating effect through the sandwich thickness, due to the evaporation energy consummation when moisture evacuates out of the sandwich sample. At the end of the pyrolysis, the hydrated sample has a residual mass fraction of about 35%, identical to that of the dry sample since the amount of residues (char) is the same. The mass loss kinetics prediction of the moisturized sandwich sample for a heat flux of 50 kW/m^2^ is well in agreement with typical TGA curves observed in the literature [34,47], allowing us to conclude that our hygro-thermal model gives very reliable prediction.

**Figure 19:**
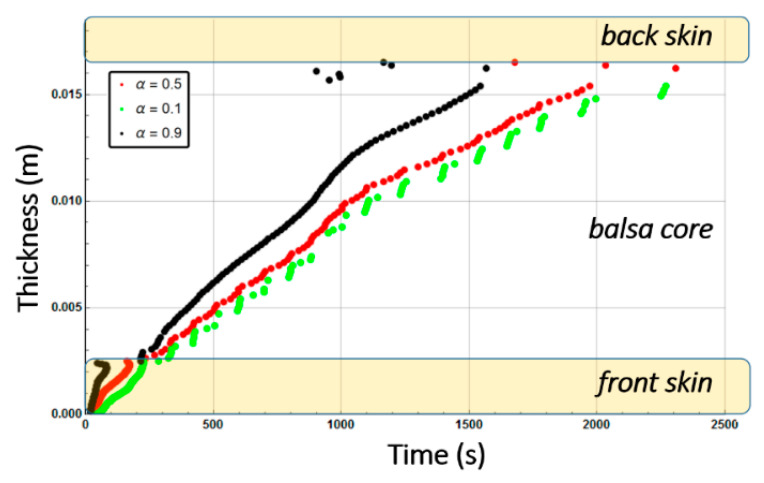
Numerical simulation prediction of the combustion advancing front for a moisturized E-glass/polyester/balsa sandwich composite material at a heat flux of 50 kW/m^2^, using the hygro-thermal model.

Post-combustion mechanical properties for the moisturized sample were predicted in a similar way to the ones for the dry sandwich composite materials. Figure 19 shows the through-thickness variation of the combustion advancing front for a glass/polyester/balsa sandwich materials exposed to a heat flux of 50 kW/m^2^. It is defined for a 50% of degradation (α(x,t)=0.5, intermediate decomposition), 10% of degradation (α(x,t)=0.9, low decomposition), and 90% of degradation (α(x,t)=0.1, almost total decomposition) for each constituent of the sandwich sample. Numerical results were obtained supposing an initial water mass fraction content of 30% related to the porosity volume fraction of the balsa core. Comparing Figure 10 and Figure 19, it could be highlighted that degradation is considerably affected by water content, due to the coupling between the evaporation process and all the others thermal phenomena. The time scales are very different with a complete degradation about twice as long for the hydrated material. It is interesting to note that the rate of degradation of the balsa core in the hydrated sample is about 0.01 mm·s^−1^ on the upper half of the core (on the heat flux side), and then 0.005 mm·s^−1^ on the lower half of the core. For the dry sample, the core degrades at a speed of about 0.03 mm·s^−1^ on its first half and 0.005 mm·s^−1^ on its lower half. The diffusion of water in the balsa thus causes a reduction by 3 of the core decomposition kinetics. Once the balsa has completely degraded on its upper half, the produced char induces the same heat shield for both samples (dry and hydrated) since the temperature reached at that time has already caused the evaporation of the water and of the infused resin throughout the core. The core decomposition kinetics is then the same (0.005 mm·s^−1^) and lasts about 1000 s.

**Figure 20:**
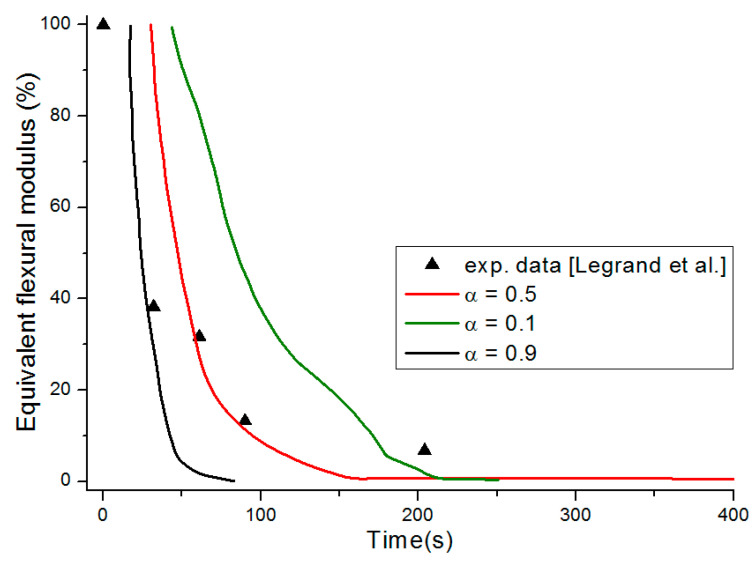
Apparent equivalent flexural modulus (contribution of only the front skin). Numerical simulation predictions as a function of time for an E-glass/polyester/balsa moisturized sandwich composite material at a heat flux of 50 kW/m^2^. Numerical predictions are superposed with the experimental data (▲) measured by Legrand et al. [29].

**Figure 21:**
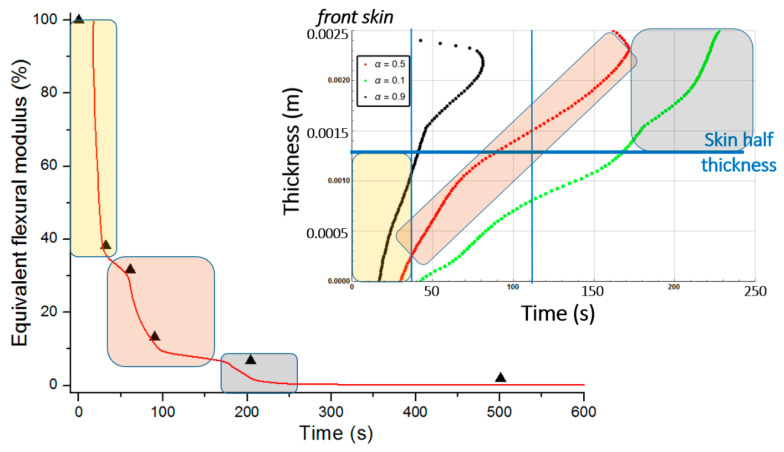
Apparent equivalent flexural modulus (contribution of only the front skin). Numerical predictions, considering a degradation rate gradient (see text for more details) as a function of time for a moisturized E-glass/polyester/balsa sandwich composite material at a heat flux of 50 kW/m^2^. Numerical predictions are superposed with the experimental data (▲) measured by Legrand et al. [29]. Insert shows prediction of the combustion advancing front in the front skin for three degradation levels α(x,t).

**Figure 22:**
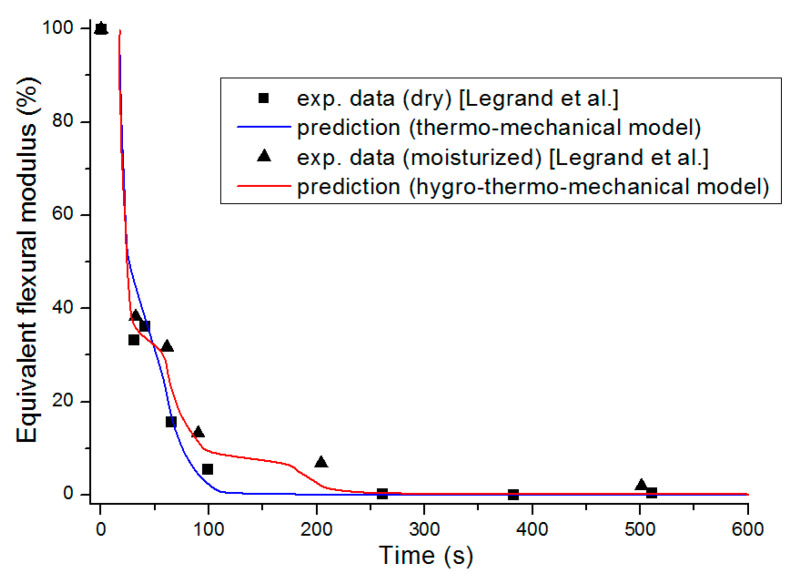
Post-combustion time dependence of the equivalent flexural modulus, considering a degradation rate gradient. Comparison of numerical prediction between thermal and hygro-thermal models for an E-glass/polyester/balsa sandwich composite at a heat flux of 50 kW/m^2^. The experimental data for both specimens (dry and moisturized) [29] are also shown.

On the basis of the evolution of the combustion progress front, the equivalent flexural modulus was calculated for the three different degradation levels (Figure 20). In relation to the dry material, the same conclusions regarding the behavior of the moisturized material at a bending stress can be given, especially since the skins for the two samples are considered dry. The time offset of the equivalent flexural modulus is a consequence of the apparent equivalent flexural modulus definition, which corresponds to the normalized flexural modulus of the front skin. For this later, thermal degradation is influenced by residual water content inside the front skin matrix initial porosity (here considered to be zero) and by water vapor flow produced by the evaporation of water content inside the balsa core, which might slightly cool the front surface. However, this influence is less important than that observed in the core and back skin because of the front skin proximity to the heat source. As for the dry sample, a flexural modulus was plotted considering a degradation rate gradient as a function of time for a moisturized E-glass/polyester/balsa sandwich composite material at a heat flux of 50 kW/m^2^ (Figure 21 and insert). The flexural modulus is then perfectly represented using this method. In Figure 22, post-combustion time dependence of the equivalent flexural modulus, considering a degradation rate gradient is shown for the moisturized and dry sandwich specimens. The influence of water content is then well observable. The developed hygro-thermal model is able to predict the post-combustion mechanical behavior of sandwich composites with polymer laminated skins and combustible core, through a relatively simple approach. It correctly takes into account the evolution of water content through the thickness of each constituent of the sandwich material. The observed effects can be important in high temperature conditions and moisture environments, and they can be estimated from the analysis of thermal degradation results, i.e., from the remaining mass fraction profile. The present hygro-thermo-mechanical model is validated in view of the very satisfying match between numerical results and experimental data.

#### 4.2.2. Glass/Vinyl Ester/Balsa Sandwich Composite

**Figure 23:**
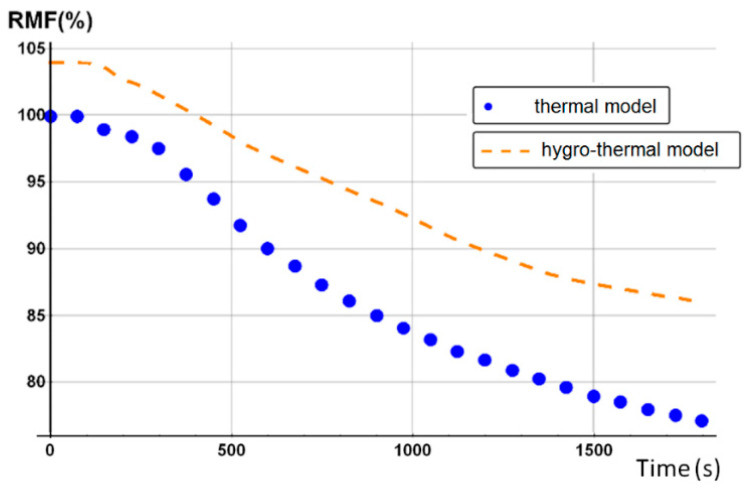
Remaining mass fraction (RMF) profile. Comparison of the numerical predictions as a function of time between thermal (blue dot line) and hygro-thermal (orange dashed line) models for an E-glass/vinyl ester/balsa sandwich composite at a heat flux of 50 kW/m^2^.

An analogous numerical study was carried out for the second material (E-glass/vinyl ester/balsa sandwich) exposed to a heat flux of 50 kW/m^2^, with an initial water mass fraction content of 20% in relation to the porosity volume fraction of the balsa core. The time profile of the total mass loss is shown in Figure 23 (dashed line) and is confronted with the prediction obtained from the previous thermal model (dot line) for a dry sample (see Figure 8). Figure 23 also shows the temperature variation as a function of time through the sandwich thickness, at five representative surfaces of the sample (upper surface of the front skin x = 0 mm, lower surface of the front skin x = 5 mm, middle surface of the core x = 20 mm, upper surface of the back skin x = 35 mm, and lower surface of the back skin x = 40 mm). Observing the RMF curves in Figure 23 and comparing the temperature variations with Figure 8, one could well highlight the importance of considering water content in the hygro-thermal model.

**Figure 24:**
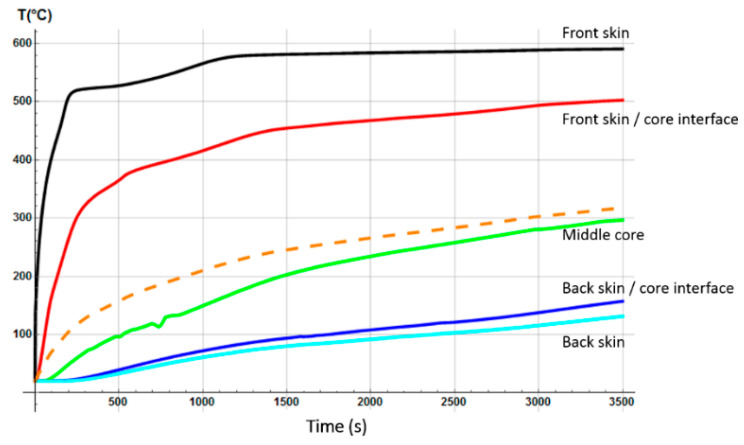
Temperature profiles prediction as a function of time (straight lines; average numerical temperature in orange dashed line) for an E-glass/vinyl ester/balsa sandwich composite, with a 20% water content in relation to the porosity volume fraction of the balsa core, at a heat flux of 50 kW/m^2^.

We immediately notice that the temperature of the front skin (Figure 24, black curve) follows approximately the same evolution as for the dry material. This seems normal since the latter is directly in contact with the incident heat flux and that it has not been considered here hydration of the skins. The differences between the wet and dry materials are observable further in depth of the material thickness, from the interface between the front skin and the balsa core. In the first 500 s of exposure to heat flux, the temperature increases rapidly up to 375 °C at the skin/core upper interface. At the same time interval, there is a mass loss of the sandwich composite of the order of 10% (Figure 23). The temperature in the balsa is then largely sufficient to induce a thermal degradation of the balsa hemicellulose and cellulose [47]. The mass loss kinetics of the material then change of slope to follow that induced by the degradation of the balsa. During this period, the resin infused into the balsa core also evaporates progressively. The temperature variation predicted in the middle of the balsa core tends to be very slow, slowed down by the fact that the balsa is hydrated. In particular a fluctuation is observed around 700 s at 110 °C, corresponding to the free and bounded water evaporation from the balsa structure. After half an hour of fire resistance at 750 °C, the temperatures at different thicknesses of the sandwich material evolve very little compared to the overall predicted variation. After a time of 3000 s (50 min), the front skin reaches a temperature of about 550 °C, the balsa core an average temperature of 300 °C and the back skin about 150 °C. In relation to the temperature reached in the three constituents of the sandwich composite material at 3000 s, the upper skin is completely decomposed into char, while the balsa core and the back skin will be partially or not degraded, respectively. Figure 23 shows finally that between the dry and hydrated materials, a gap of 10% in mass loss in favor of the hydrated material is preserved almost throughout the combustion. Water content works as a thermal barrier giving an isolating effect through the sandwich thickness, due to the evaporation energy consummation when moisture evacuates out of the sandwich sample, as previously shown for the E-glass/polyester/balsa sandwich.

**Figure 25:**
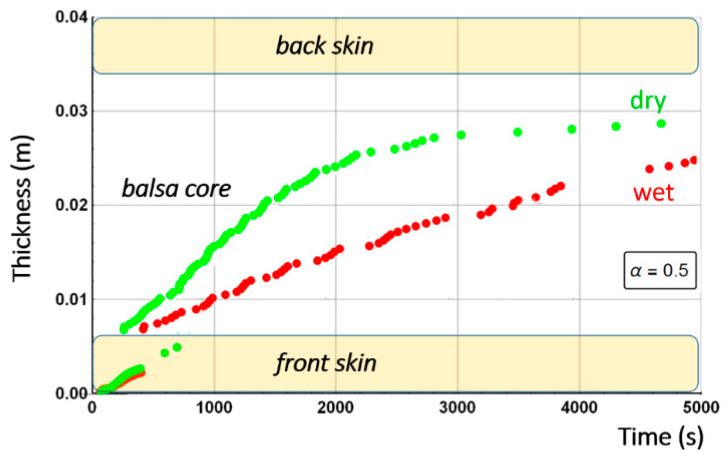
Prediction of the combustion advancing front for a moisturized E-glass/vinyl ester/balsa sandwich composite material (wet, red curve) at a heat flux of 50 kW/m^2^, with a 20% water content in relation to the porosity volume fraction of the balsa core using the hygro-thermal model. Prediction comparison of the combustion advancing front for the same dry material (green curve).

Post-combustion mechanical properties for the moisturized sample were predicted in a similar way to the ones for the dry sandwich composite material. As shown previously, water content alters the thermal answer during heat flux exposition (fire) and therefore directly influences the post-combustion mechanical properties. Figure 25 shows the combustion advancing front prediction defined for a degradation rate of 50%, for a moisturized E-glass/vinyl ester/balsa sandwich composite material having a 20% water content in relation to the porosity volume fraction of the balsa core. For comparison, Figure 25 also includes the combustion advancing front prediction for the same dry material (0% water content). It could be highlighted that degradation is considerably affected by water content, due to the coupling between the evaporation process and all the others thermal phenomena. It is interesting to observe that the balsa core is degraded with very different behavior. Indeed, for the dry material, it could be highlighted a change in slope around 1500 s of exposure time to the heat flux, in correspondence with a gradual rise in temperature to about 300 °C in the middle of the core. For this degradation time, the material has already lost a large part of its mass, and the temperature is now sufficient to largely degrade the hemicellulose and cellulose, and to induce the degradation of the lignin contained in the balsa [47]. From a chemical point of view, the balsa core then enters into the phase of passive pyrolysis in which the lignin decomposes very slowly. As for the hydrated material, it decomposes much more slowly and in a progressive and constant manner. Of course this is also related to the predicted temperature variation in the core. After 1500 s of exposure, we observe a gap of 25% of the position of the combustion advancing front, which is maintained around 3500 s. For a very long time (>5000 s), the internal temperature of balsa in its different thicknesses are almost identical for both samples (dry and hydrated), and the two curves tend to overlap, inducing a similar amount of residues (char). It is interesting to note that the kinetics of decomposition of the balsa core, on the upper half of the core (heat flux side), of the wet material is about 0.005 mm·s^−1^, and about 0.01 mm·s^−1^ for the dry one.

**Figure 26:**
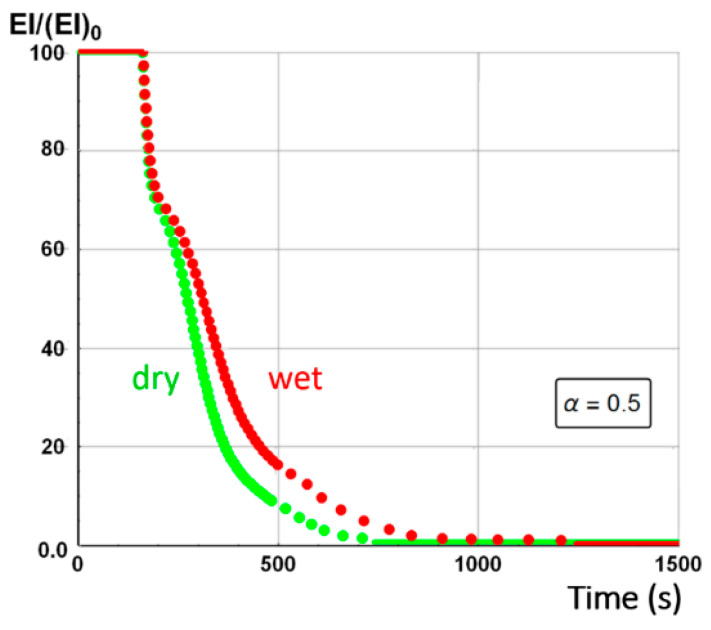
Post-combustion time dependence of the normalized flexural modulus. Comparison of numerical predictions as a function of combustion time between pure thermal (dry sample, green curve) and hygro-thermal (wet sample, red curve; 20% water content in relation to the porosity volume fraction of the balsa core) models for an E-glass/vinyl ester/balsa sandwich composite at a heat flux of 50 kW/m^2^ and for a degradation level of 50%.

Having predicted the variation of the combustion advancing front in the wet material, it is possible to calculate the equivalent flexural modulus (Figure 26) at a heat flux of 50 kW/m^2^ and for a degradation level of 50%. We observe the same tendency for both sample (dry and wet), with slightly longer ruins in the case of the hydrated material. Remember here that the skins for the two samples are considered without water diffusion. The observed difference in Figure 26 is then not due to the residual water content in the front skin but induced by the water vapor flow produced during water evaporation contained inside the balsa, which might slightly cool the front interface and so the front skin. The combustion advancing front in the front skin is, therefore, slightly slower in the case of the wet specimen (Figure 25). The complete loss of flexural strength occurs with an additional delay of 200 s in favor of the hydrated material. The developed advanced hygro-thermo-mechanical model correctly takes into account the evolution of water content inside each constituent of the sandwich material.

## 5. Conclusions

Does water content alter the thermo-mechanical behavior and properties of sandwich composite material during a fire scenario?

The thermal decomposition of sandwich composite materials can be adequately predicted thanks to numerical models, such as the presented one-dimensional hygro-thermal model of Legrand et al. The present numerical model also allows to accurately predict the post-combustion mechanical properties for sandwich composites having polymer laminated skins and combustible core. The results well underline the effects related to the presence of water content field within the sandwich composite material, sometimes inducing a large change in the evolution of key parameters, such as the mass loss and the combustion advancing front. Of course, the greater the heat flux at the surface of the material, and with a large amount of water diffused into the skins and the core, the more the behavior of the material and its properties are modified, for each component (skins and core) as a function of time and also of the thickness. Thus, the use of the proposed model makes it possible to successfully predict these thermal behaviors at both the components and material scales, in a large variety of scenarios (value of the heat flux, ambient conditions of temperature and pressure, etc.) and sandwich materials (geometry, nature of the resin and the core, type of reinforcing fibers, nature of the resin infused into the core, hydration rate, porosity rate, etc.).

An extension to a 2D or 3D model could be formulated in order to take into account for spatial different properties and mechanisms, such as conductivity and expansion, depending on the composite material direction. Post-combustion mechanical properties are estimated by a combination of a bi-layer model (char and virgin) for each constituent of the sandwich material. Therefore, they depend directly on the thermal response. Even if a good agreement between experimental and numerical data has been found here, it is envisaged to improve the model in order to consider the complexity of sandwich materials mechanical phenomena, such as damage and failure. These phenomena could be especially important for thinner composites skins and for interfaces between skins and the core, which are not modeled in the presented model interfaces modeling would enable to more accurately predict post-combustion mechanical performances during first steps of mechanical degradation, even before any considerable thermal decomposition. That requires to propose new mechanical models to know the material stress-strain state, including explicit temperature dependences on material behavior and mechanical properties. Thus, a change to the ply scale modeling is recommended. This approach will allow to analyze not only post-combustion performances but also more accurate thermo-mechanical response during the material exposition to the heat source. Despite that, and as indicated, the presented model is in very acceptable agreement since thermal decomposition starts, which represent a critical phenomenon for naval and aeronautics materials.

The highlight of the presented model is the ability to play on the amount of water present in the constituents of the material in order to accurately predict the thermal response for wet or humid materials in a fire scenario. Indeed, left in normal conditions of pressure and temperature, the balsa already picks up between 8% and 15% by weight of water. If this sample is then used in the manufacture of sandwich plate, the finalized material is hydrated and its thermo-mechanical behavior has changed. Later, depending on its use and the environment, the water content of this sandwich material can still evolve. Further research should ongoing to better describe the influence of moisture on the thermal properties, such as heat capacity, conductivity, permeability, or thermal expansion coefficients, and mechanical properties, such as ply and interfaces stiffness and strengths. Thus, future works must focus on the analysis about how the water content affects not only the thermal response but also the mechanical one, which seems necessary to better understand different failures modes and durability of composites materials in extreme conditions. Particularly in the marine or aeronautical industries, materials are subject to large variations of water content and temperature. In addition, because of the flexibility of the model, this study is part of the investigation of the thermal and hygroscopic durability of materials.

## Figures and Tables

**Table 2:** Material properties for E-glass/vinyl ester composite laminate and balsa core.

Property	Values		References	
	Glass/Vinyl Ester	Balsa	Glass/Vinyl Ester	Balsa
Fraction volume of fibers (-) V_f_	0.55	-	[34]	
Kinetics rate constant (1/s) A	2 × 10^13^	6.7 × 10^7^	[34]	[34]
Activation energy (J/mol) E_a_	212,000	116,488	[34]	[34]
Reaction order (-) n	1	1	[34]	[34]
Remaining matrix Mass Fraction (-) α	0.03	0.01	[34]	[34]
Heat of decomposition (J/kg) Q_p_	378,800	556,000	[34]	[34]
Density of glass balsa core (kg/m^3^) ρ_balsa_	-	150		[34]
Density of glass fiber (kg/m^3^) ρ_fiber_	2560	-	[34]	
Density of vinyl ester (kg/m^3^) ρ_matrix_	1140	-	[34]	
Thermal conductivity of balsa core (W/(m K)) k_balsa_	-	0.2		[34]
Thermal conductivity of glass fiber (W/(m K)) k_fiber_	1.09	-	[34]	
Thermal conductivity of vinyl ester (W/(m K)) k_matrix_	0.19	-	[34]	
Specific heat of glass fiber (J/(kg K)) c_p,fiber_	760	-	[34]	
Specific heat of vinyl ester (J/(kg K)) c_p,matrix_	1509	-	[34]	
Specific heat of gas for balsa core (J/(kg K)) c_pg,balsa_	-	1009		[34]
Specific heat of gas for vinyl ester (J/(kg K)) c_pg,matrix_	2387	-	[34]	
Thickness of the skin and balsa core (m) L	5 × 10^−3^	30 × 10^−3^	[34]	[34]
Virgin coefficient of linear thermal expansion [1/K] α_v_	2.52 × 10^−5^	3 × 10^−5^	[46]	[42]
Char coefficient of linear thermal expansion [1/K] α_c_	6.3 × 10^−5^	0	[46]	[42]
Virgin material permeability [m^2^] γ_v_	8.29 × 10^−17^	9 × 10^−12^	[46]	[46]
Char material permeability [m^2^] γ_c_	1.56 × 10^−10^	8.7 × 10^−12^	[46]	[46]
Molecular weight of gases [kg/mol] M	18.35 × 10^−3^	18.35 × 10^−3^	[18]	[46]
Room temperature [°C] T_∞_	20	-		
Room pressure [Pa] P	101,325	-		
Pressure on the back surface [Pa] P_bs_	101,325	-		

## Nomenclature

A	Pre-exponential factor of Arrhenius law (s^−1^)
c_i_	Specific heat of state i (J·kg^−1^·K^−1^)
c_p_	Specific heat of the material (J·kg^−1^·K^−1^)
c_pg_	Specific heat of gas (J·kg^−1^·K^−1^)
$c_{pvH_{2}O}$	Specific heat of water vapor (J·kg^−1^·K^−1^)
d	Total thickness (m)
d_c_	Carbonized layer thickness (m)
d_n_	Balance bending forces’ thickness (m)
E_a_	Activation energy (J·mol^−1^)
E	Young modulus (MPa)
<EI>	Flexural modulus (MN·m^2^)
f_c_	Correction factor (-)
h_g_	Enthalpy of pyrolysis gases (J·kg^−1^)
h_s_	Enthalpy of the solid material (J·kg^−1^)
h_conv_	Convective heat transfer coefficient (W·m^−2^·K^−1^)
I	Quadratic moment (m^4^)
k_g_	Thermal conductivity of volatile gases (W·m^−1^·K^−1^)
k_i_	Thermal conductivity of state i (W·m^−1^·K^−1^)
k_x_	Thermal conductivity of solid material (W·m^−1^·K^−1^)
L	Thickness of material (m)
m_g_	Volatile gases mass (kg)
${\dot{m}}_{g}$	Mass flow rate per unit area of pyrolysis gases through the reaction zone (kg·s^−1^·m^−2^)
m_m_	Mass of matrix (kg)
m_s_	Solid mass of material (kg)
$m_{H_{2}O}$	Water mass (kg)
M	Average molecular weight of gases (kg·mol^−1^)
n	Order of decomposition reaction
P	Internal pressure of gases (atm)
$q_{radiation}^{\prime \prime }$	Heat flux (W·m^−2^)
Q_p_	Heat of decomposition (J·kg^−1^)
R	Gas constant (8.314 J·mol^−1^·K^−1^)
t	Time (s)
T	Temperature (K)
T_∞_	Ambient temperature (K)
V_f_	Volume fraction of fibers (%)
V_m_	Volume fraction of the matrix (%)
x	Though-thickness coordinate (m)
X	Mass fraction (-)
α	Remaining fraction of virgin material (-)
α_i_	Linear thermal expansion coefficient of state i (K^−1^)
γ	Permeability of material (m^2^)
Δh_fg_	Latent heat of water (J·kg^−1^)
Δh_l_	Evaporation heat of free liquid water (J·kg^−1^)
Δh_desorp_	Evaporation heat of bound water (J·kg^−1^)
ψ_i_	Permeability coefficient of state i (K^−1^)
ε_m_	Material surface emissivity (-)
ε_s_	Source emissivity (-)
ζ	Dimensionless expansion factor (-)
η	Dimensionless permeability factor (-)
μ	Gases viscosity (Pa·s)
ρ	Material density (kg·m^−3^)
σ	Stefan-Boltzmann constant (5.67 × 10^8^ W·m^−2^·K^−4^)
Φ	Porosity of material (-)
**Subscripts**	
_a_	Activation
_c_	Carbonized state
_composite_	Composite
_conv_	Convection
_0_	Initial state
_f_	Final state
_fiber_	Fiber
_fsp_	Fiber saturation point
_g_	Gas
$H_{2}O$	Water
_inf_	Inferior
_m_	Matrix
_p_	Pyrolysis
_s_	Solid, Source
_sat_	Saturation
_sup_	Superior
v	Virgin state
$vH_{2}O$	Water vapor
